# A New Ankylosaurid Dinosaur from the Upper Cretaceous (Kirtlandian) of New Mexico with Implications for Ankylosaurid Diversity in the Upper Cretaceous of Western North America

**DOI:** 10.1371/journal.pone.0108804

**Published:** 2014-09-24

**Authors:** Victoria M. Arbour, Michael E. Burns, Robert M. Sullivan, Spencer G. Lucas, Amanda K. Cantrell, Joshua Fry, Thomas L. Suazo

**Affiliations:** 1 Department of Biological Sciences, University of Alberta, Edmonton, Alberta, Canada; 2 New Mexico Museum of Natural History and Science, Albuquerque, New Mexico, United States of America; 3 Department of Geosciences, Fort Hays State University, Hays, Kansas, United States of America; Raymond M. Alf Museum of Paleontology, United States of America

## Abstract

A new ankylosaurid (Ankylosauria: Dinosauria), *Ziapelta sanjuanensis*, gen. et sp. nov., is based on a complete skull, an incomplete first cervical half ring, a possible fragment of the second cervical half ring, and additional fragmentary osteoderms. The holotype specimen is from the Upper Cretaceous (Upper Campanian, Kirtlandian Land-Vertebrate Age) Kirtland Formation (De-na-zin Member) at Hunter Wash, San Juan Basin, in northwestern New Mexico, USA. Diagnostic characters of *Ziapelta* include: a large, prominent triangular median nasal caputegulum; a mixture of flat and bulbous frontonasal caputegulae; ventrolaterally oriented squamosal horns with a sharp, prominent dorsal keel; and the ventral surface of basicranium with three prominent anteroposteriorly oriented fossae. A phylogenetic analysis suggests that *Ziapelta* is not closely related to the other ankylosaurid from the De-na-zin Member, *Nodocephalosaurus*, but allies it to the northern North American ankylosaurids *Ankylosaurus*, *Anodontosaurus*, *Euoplocephalus*, *Dyoplosaurus*, and *Scolosaurus*.

## Introduction

The terrestrial deposits in the San Juan Basin of northwestern New Mexico have produced significant specimens of dinosaurs, representing most of the major groups known in the North American Upper Cretaceous [1], [2]. Ankylosaurid remains from these beds, largely consisting of isolated osteoderms, vertebrae and some fragmentary appendicular elements, have long been recognized in the Fruitland and Kirtland formations [3], [4]. These incomplete specimens were generally referred to North American taxa that are better known from Montana and Alberta [4], [5]. More recent discoveries have shown that the ankylosaurids of New Mexico were distinct from those of Montana and Alberta.

*Nodocephalosaurus kirtlandensis* Sullivan, 1999 [6] was named from the De-na-zin Member of the Kirtland Formation based on a partial skull. Because it was the only diagnosable ankylosaurid known from the De-Na-Zin Member, all ankylosaurid material collected subsequently was referred to that taxon. Other specimens referred to *Nodocephalosaurus* included isolated cranial osteoderms, a cervical osteoderm, two free caudal vertebrae, and several partial tail club knob osteoderms [7], [8].

*Glyptodontopelta mimus* Ford, 2000 [9] was named based on a fragment of the pelvic shield and other isolated osteoderms from the overlying Maastrichtian Naashoibito Member of the Ojo Alamo Formation in the San Juan Basin. Burns [10] supported the validity of *Glyptodontopelta* and reclassified it as a nodosaurid, also synonymizing *Edmontonia australis* Ford, 2000 [9], with *Glyptodontopelta mimus*. Known only from osteoderms, *Glyptodontopelta* is currently the only nodosaurid recognized from the San Juan Basin.

More recently, Burns and Sullivan [11] named a small ankylosaurid *Ahshislepelta minor*, Burns and Sullivan, 2011, from the Hunter Wash Member of the Kirtland Formation. The holotype includes a partial left humerus, right and left partial scapulocoracoids, numerous vertebral fragments, and complete and fragmentary thoracic osteoderms. The taxon is diagnosed on the dorsolateral overhang of the scapular acromion process to 25% of the dorsoventral width of the scapula. In addition, its osteoderm surface texture is characterized by uniformly distributed pitted rugosity, and sparse distribution of reticular neurovascular grooves with neurovascular foramina extending perpendicularly to obliquely into the bone [11], [12]. *Nodocephalosaurus* osteoderms have a more prominent, projecting rugosity [12]. The surface texture of *Ahshislepelta* osteoderms distinguishes it from contemporaneous ankylosaurids with the exception of some specimens of *Euoplocephalus tutus* (Lambe, 1902) [13] (*sensu stricto*; [14]).

**Figure 1:**
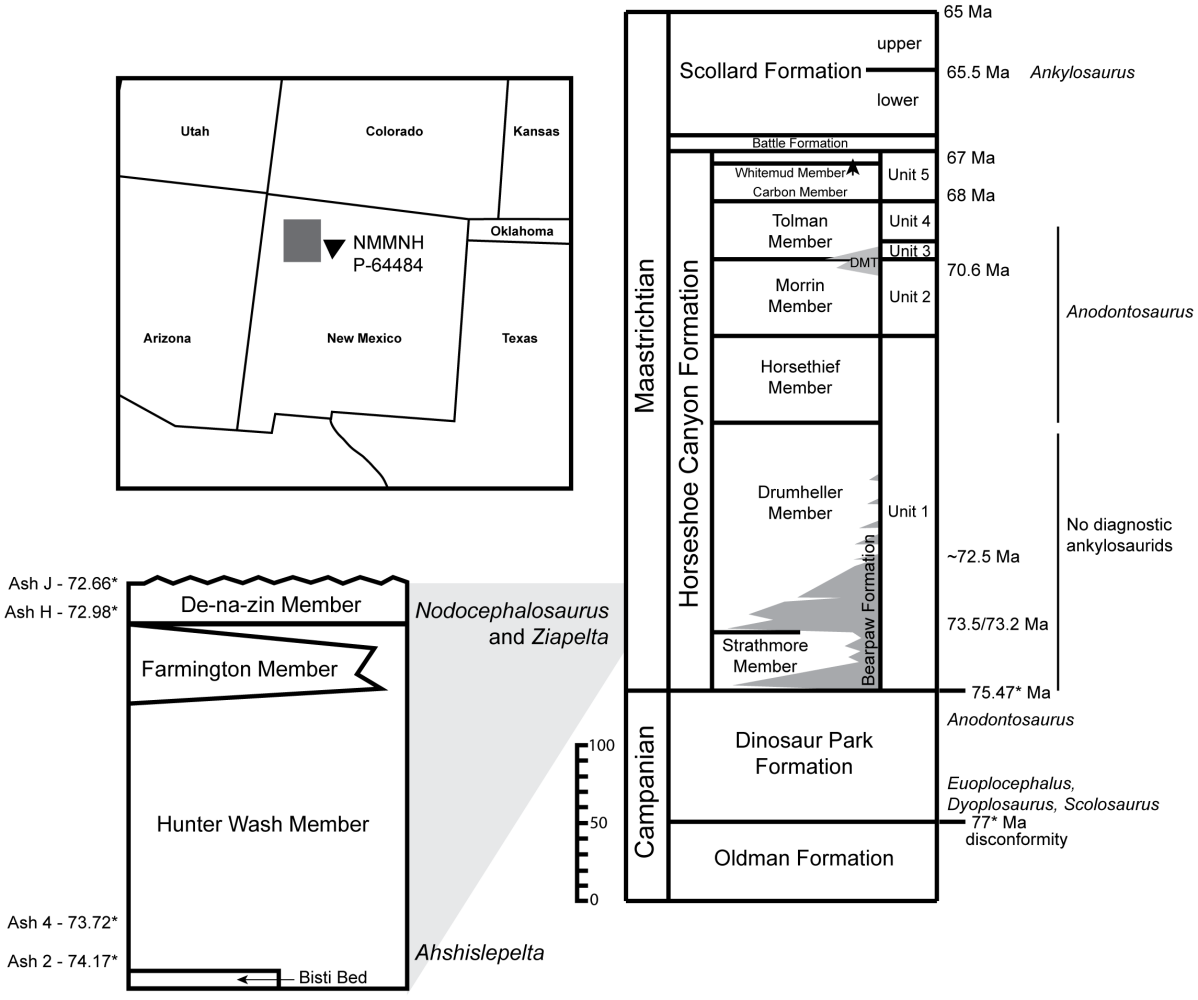
Locality map, San Juan Basin, New Mexico, southwest USA. Stratigraphic position of *Ziapelta sanjuanensis*, gen. et sp. nov., in the De-na-zin Member, Kirtland Formation, and comparison with the stratigraphic positions of other ankylosaurids in New Mexico and Alberta. Alberta stratigraphic column modified from Eberth and Braman [56]. Dates with asterisks represented revised dates from Roberts et al. [16].

In 2011, a team led by R. M. Sullivan from the SMP and NMMNH (see Table 1) collected an ankylosaurid skull and incomplete first and second cervical half rings from the De-na-zin Member of the Kirtland Formation. This specimen, NMMNH P-64484, was collected stratigraphically low in the De-na-zin Member of the Kirtland Formation, approximately 6–10.5 meters below, and 525 meters from, the type locality of the ankylosaurid *Nodocephalosaurus kirtlandensis*, in the region of the east fork of Hunter Wash (Fig. 1). The specimen (skull, incomplete first and second cervical half rings, and miscellaneous osteoderms) was discovered semi-articulated, with the ventral side up, in a weathered, nearly unconsolidated light grayish-tan silty sandstone. No other elements were uncovered, suggesting that the head and cervical armor were separated from the body prior to burial.

**Table 1:** Institutional abbreviations.

Institutional Abbreviation	Institution Name and Location
**AMNH**	American Museum of Natural History, New York, New York, USA;
**NMMNH**	New Mexico Museum of Natural History and Science, Albuquerque, New Mexico, USA;
**ROM**	Royal Ontario Museum, Toronto, Ontario, Canada;
**SMP**	State Museum of Pennsylvania, Harrisburg, Pennsylvania, USA;
**UALVP**	University of Alberta Laboratory for Vertebrate Paleontology, Edmonton, Alberta, Canada;
**UMNH**	Natural History Museum of Utah, Salt Lake City, Utah;
**USNM**	National Museum of Natural History, Washington, DC, USA

The Kirtland Formation (Fig. 1) consists of interbedded sandstone, siltstone, mudstone, coal and shale and is up to 594 m thick locally [15]. Sullivan and Lucas [1] recognized three members within the formation: Hunter Wash, Farmington, and De-na-zin. The uppermost De-na-zin Member is overlain by an unconformity that marks the lower boundary of the lower conglomerate of the Ojo Alamo Formation [1]. To date, two volcanic ashes with published ^40^Ar/^39^Ar ages are known from the De-na-zin Member: Ash H, dated at 72.98±0.18 Ma and Ash J, dated at 72.66±0.25 Ma (dates represent recent recalibrations by Roberts et al. [16]; see also [1], [17]–[19]). NMMNH P-64484 was found approximately 12 m below Ash H, based on physical correlation over a distance of 5.1 km.

NMMNH P-64484 represents a new ankylosaurid taxon that can be differentiated from the coeval *Nodocephalosaurus kirtlandensis*. It is also distinct from the Campanian-Maastrichtian ankylosaurids from northern Laramidia, including *Euoplocephalus tutus* and *Dyoplosaurus acutosquameus* Parks, 1924 [20] from the Dinosaur Park Formation of Alberta, *Scolosaurus cutleri* Nopcsa, 1928 [21] ( = *Oohkotokia*, Penkalski, in press [22]) from the Dinosaur Park Formation of Alberta and Two Medicine Formation of Montana, *Anodontosaurus lambei* Sternberg, 1929 [23] from the Horseshoe Canyon Formation of Alberta, and *Ankylosaurus magniventris* Brown, 1908 [24] from the Scollard Formation of Alberta, the Hell Creek Formation of Montana, and the Lance Formation of Wyoming [14], [25].

## Materials and Methods

The specimen (NMMNH P-64484) consists of a complete skull, the left side of the first cervical half ring, fragmentary second cervical half ring, and numerous fragmentary postcranial osteoderms. It was found in 2011 in the Bisti/De-na-zin Wilderness by R. M. Sullivan and was collected by R. M. Sullivan, J. Fry, A. K. Cantrell and T. L. Suazo, and is reposited at the New Mexico Museum of Natural History and Science in Albuquerque, New Mexico, USA.

External variation in morphology among Late Cretaceous ankylosaurid specimens was noted through measurements, observations, and photographs [File S1]. All measurements were taken with digital calipers or flexible measuring tape. The stratigraphic nomenclature and age assignments of the San Juan Basin Upper Cretaceous strata follow Sullivan and Lucas [1]. Nomenclature for ankylosaurid cranial ornamentation follows Arbour and Currie [14].

The phylogenetic position of *Ziapelta* within the Ankylosauridae was assessed via a cladistic parsimony analysis of 154 characters and 19 taxa [File S1, File S2] including 12 ingroup taxa and the outgroup taxa *Lesothosaurus diagnosticus* Galton, 1978 [26] (a basal ornithischian), *Scelidosaurus harrisonii* Owen, 1861 [27] (a basal thyreophoran or basal ankylosaur), *Stegosaurus* Marsh, 1877 [28] (a stegosaur), *Panoplosaurus mirus* Lambe, 1919 [29], and *Pawpawsaurus campbelli* Lee, 1996 [30], (nodosaurid ankylosaurs), and *Gastonia burgei* Kirkland, 1998 [31] (a basal ankylosaur or basal ankylosaurid). Although *Ahshislepelta* is also known from New Mexico, it was not included in the phylogenetic analysis because only 12 characters (those related to the scapula and humerus) could potentially be coded. The character matrix [File S2] was updated from Arbour et al. [32]; changes to characters and character codings are noted in [File S1]. All characters were treated as unordered and of equal weight. The data matrix was analyzed using TNT [33], with the tree bisection reconnection (TBR) swapping algorithm and 1000 replications. Bootstrap values (using 1000 replicates) were found via a heuristic search of 1000 replicates with a random addition sequence. An analysis for safe taxonomic reduction was performed using TAXEQ3 [34]. To determine the minimum tree length if *Nodocephalosaurus* and *Ziapelta* were constrained as sister taxa, the tree file was manually edited such that *Nodocephalosaurus* and *Ziapelta* formed a clade. These taxa were then defined as a group in TNT, and the traditional search was run again with constraints enforced.

### Ethics Statement

The specimen was collected on federal/public protected land known as the Bisti/De-na-zin Wilderness Study Area in New Mexico, under the United States Department of the Interior, Bureau of Land Management, Paleontological Resources Use Permit number NM11-004S NLCS BDNZ, issued 13 April 2011 and valid from 1 May 2011 through 1 May 2014. All necessary permits were obtained for the described study, which complied with all relevant regulations.

### Nomenclatural Acts

The electronic edition of this article conforms to the requirements of the amended International Code of Zoological Nomenclature, and hence the new names contained herein are available under that Code from the electronic edition of this article. This published work and the nomenclatural acts it contains have been registered in ZooBank, the online registration system for the ICZN. The ZooBank LSIDs (Life Science Identifiers) can be resolved and the associated information viewed through any standard web browser by appending the LSID to the prefix “http://zoobank.org/”. The LSIDs for this publication are: urn:lsid:zoobank.org:pub:3401469D-AB4A-4D09-9884-F547215ACD54, urn:lsid:zoobank.org:act:8FC0324E-C18B-4CD6-830B-92C75AA32A58, and urn:lsid:zoobank.org:act:96AA0FCF-D7E4-438B-9B1E-FB9F3B6532F0. The electronic edition of this work was published in a journal with an ISSN, and has been archived and is available from the following digital repositories: PubMed Central, LOCKSS.

## Systematic Paleontology

**Dinosauria Owen, 1842 [35]**

**Ornithischia Seeley, 1888 [36]**

**Thyreophora Nopcsa, 1915 [37]**

**Ankylosauria Osborn, 1923 [38]**

**Ankylosauridae Brown, 1908 [24]**

***Ziapelta*** **gen. nov. urn:lsid:zoobank.org:act:8FC0324E-C18B-4CD6-830B-92C75AA32A58**

**Type and only known species.** *Ziapelta sanjuanensis*.

**Etymology.** *Zia*, after the Zia sun symbol, a stylized sun with four groups of rays, having religious significance to the Zia people of New Mexico, and the iconic symbol on the state flag of New Mexico; *pelta* (Latin), a small shield, in reference to the osteoderms found on all ankylosaurids.

**Diagnosis.** Same as for species.

***Ziapelta sanjuanensis*** **sp. nov. urn:lsid:zoobank.org:act:96AA0FCF-D7E4-438B-9B1E-FB9F3B6532F0**

**Etymology.** In reference to San Juan County and the structural basin from which the specimen was derived.

**Figure 2:**
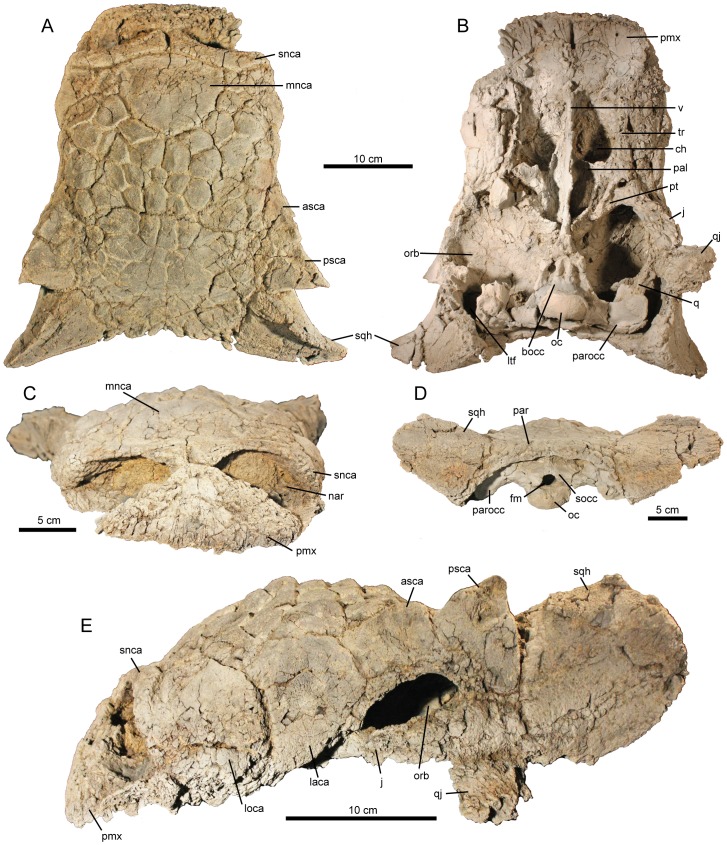
*Ziapelta sanjuanensis*, gen. et sp. nov., (holotype NMMNH P-64484), complete skull. A, dorsal view; B, ventral view; C, anterior view; D, occipital view; and E, left lateral view. Abbreviations: asca, anterior supraorbital caputegulum; bas, basioccipital; ch, choana; fm, foramen magnum; j, jugal; laca, lacrimal caputegulum; loca, loreal caputegulum; ltf, laterotemporal fenestra; mnca, median nasal caputegulum; nar, external naris; oc, occipital condyle; orb, orbit; pal, palatine; par, parietal; parocc, paroccipital process; pmx, premaxilla; psca, posterior supraorbital caputegulum; pt, pterygoid; q, quadrate; qj, quadratojugal; qjh, quadratojugal horn; snca, supranarial caputegulum; socc, supraoccipital; sqh, squamosal horn; tr, tooth row; v, vomer.

**Figure 3:**
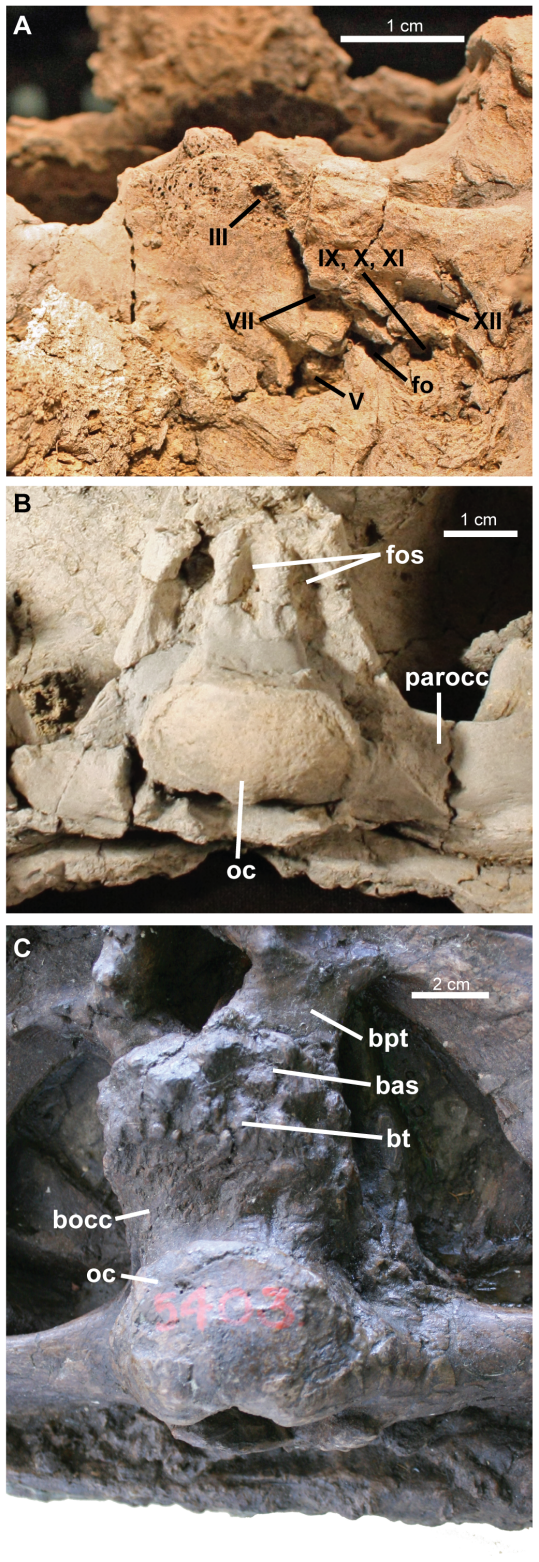
*Ziapelta sanjuanensis*, gen. et sp. nov., (holotype NMMNH P-64484), basicranium, in A, left lateral view (anterior is to the left, dorsal is towards the bottom), and B, ventral view (anterior is towards the top). AMNH 5403, *Euoplocephalus tutus*, basicranium in C, ventral view (anterior is towards the top). Abbreviations: III, opening for the oculomotor nerve; VII, opening for the facial nerve; V, opening for the trigeminal nerve; IX-X-XI, opening for the glossopharyngeal, vagus, and accessory nerves; XII, opening for the hypoglossal nerve; bas, basisphenoid; bocc, basioccipital; bpt, basipterygoid process; bt, basal tubera; fo, foramen ovale; fos, fossa in basioccipital; oc, occipital condyle.

**Figure 4:**
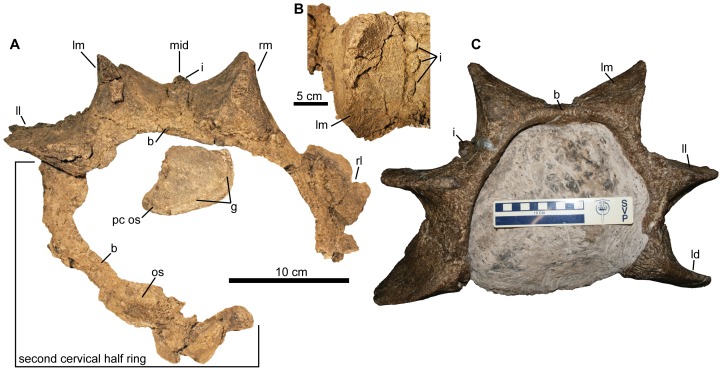
Cervical half rings of *Ziapelta sanjuanensis*. A) Incomplete first and second cervical half rings of NMMNH P-64484 (holotype), with isolated post-cervical osteoderm, as preserved in situ. First cervical half ring is in posterior view. B) Dorsal view of left medial osteoderm showing smaller interstitial osteoderms, anterior is up. C) Isolated first cervical half ring NMMNH P-66930 (referred specimen) in anterior view. Abbreviations: b, band; g, groove; i, interstitial osteoderm; ld, left distal osteoderm; ll, left lateral osteoderm; lm, left medial osteoderm; mid, midline of the cervical half ring; os, osteoderm; pc os, post-cervical osteoderm; rl, right lateral osteoderm; rm, right medial osteoderm.

**Holotype.** NMMNH P-64484 (Figs. 2–4a,b), complete skull, left side of first cervical half ring, fragmentary second cervical half ring, and numerous fragmentary postcranial osteoderms.

**Holotype Locality.** NMMNH L-8514. East branch of Hunter Wash, San Juan County, New Mexico, USA.

**Stratigraphic horizon and age.** De-na-zin Member, Kirtland Formation; Upper Campanian, Upper Cretaceous).

**Referred specimens.** NMMNH P-66930, a complete first cervical half ring collected from the Hunter Wash Member of the Kirtland Formation, Bisti/De-na-zin Wilderness, approximately 8.4 km west from the holotype specimen.

**Diagnosis.** *Ziapelta* is unique among ankylosaurids in having the following features: prominent, large, sub-triangular median nasal caputegulum; dorsoventrally deep squamosal horns curved anteriorly at the tips; three deep fossae on the ventral surface of the basicranium. Differs from other ankylosaurids in possessing a mixture of flat and weakly bulbous frontonasal caputegulae. Differs from *Nodocephalosaurus kirtlandensis* in the irregular basal shape of the frontonasal caputegulae and in the manner in which the caputegulae are bulbous (conical in Nodocephalosaurus, irregularly convex in Ziapelta), and the shape of the squamosal horns, which have a sharper, more prominent dorsal keel. Distal osteoderm of the cervical half ring does not envelop the terminus of the underlying bony band, unlike the condition in *Ankylosaurus magniventris*, *Anodontosaurus lambei*, *Euoplocephalus tutus*, and *Scolosaurus cutleri*.

## Osteological Description

### Skull

The skull (Fig. 2) is well preserved and nearly complete, with minimal dorsoventral compression. The ventral surface is fractured, particularly on its anterior half. In dorsal view, the skull (excluding the squamosal and supraorbital horns) has a sub-rectangular outline. The lateral sides of the snout are parallel in dorsal view, and there is no constriction anterior to the orbit. The widest point of the skull is at the posterior tips of the squamosal horns. In lateral view, the skull has an arched profile, with a convex dorsal surface between the orbits and nares, and a flat dorsal surface between the orbits and nuchal shelf. The left side of the skull is more complete than the right, preserving part of the ventral border of the orbit and a remnant of the left quadratojugal horn.

### Rostral Region

In ventral view, the paired premaxillae form a broad, square outline (Fig. 2B), and the anterior margins of the premaxillae are eroded. The boundaries of the maxillae are obscured by cranial ornamentation. The supranarial caputegulae form the dorsal border of the nasal vestibule (Fig. 2A), which is ovoid in anterior view (Fig. 2C). They are dorsoventrally tallest near the midline of the skull, and are separated at the midline by a 0.9 cm gap. These caputegulae are arched, with the peak of the arch located at about three-quarters of the length of the caputegulum from the median sagittal plane. The supranarial caputegulae have a smooth texture, similar to the other cranial caputegulae.

Both maxillae are preserved, but are fractured and weathered. The maxillae articulate with the premaxillae anteriorly and the ectopterygoids posteriorly. In lateral view, each maxilla is mostly covered by a large, flat, rectangular loreal caputegulum, which extends onto the dorsum of the skull posterior to the supranarial caputegulae (Fig. 2E). Posterior to the loreal caputegulum and anterior to the orbit is the flat, square lacrimal caputegulum, which does not extend as far onto the dorsal surface of the skull as does the loreal caputegulum. The lacrimal caputegulum is anteroposteriorly longer than the loreal caputegulum, with a ventral length of 6.6 cm. The ventral margin of the orbit is formed by the lacrimal and jugal, but this region is damaged in NMMNH P-64484, so the contact between these bones is not visible.

Posterior to the supranarial caputegulae is a relatively large, flat, triangular median nasal caputegulum, which has a maximum width of 11.3 cm and a maximum length along the midline of 9.2 cm (Fig. 2A). Overall, the pattern of caputegulae is bilaterally symmetrical. A nearly bilaterally symmetrical mosaic of smaller caputegulae covers the nasals and frontals, and obscures the contact between these bones. These frontonasal caputegulae are rectangular to hexagonal and 2.4 to 3.5 cm in diameter. These caputegulae form irregular sub-parasagittal rows. Frontonasal caputegulae towards the anterior and lateral sides of the skull are flat or concave, whereas frontonasal caputegulae along the midline and more posterior areas of the skull are convex. Shallow grooves between the frontonasal caputegulae make each caputegulum distinct from adjacent caputegulae.

### Temporal Region

The boundaries between individual caputegulae become less distinct towards the frontoparietal region of the skull, and the boundary between the frontals and parietals is not visible (Fig. 2A). Caputegulae are most distinct laterally, and less distinct towards the midline of the skull in this region. Two laterally keeled supraorbital caputegulae overhang the orbit (Fig. 2A). A shallow notch separates the peak of each supraorbital. Both are triangular in dorsal view. The posterior supraorbital caputegulum is sharp and prominent, and the anterior supraorbital is less prominent. The anterior supraorbital is 6 cm long. The dorsal surface of the posterior supraorbital is concave.

Posterior to the supraorbitals, the parietals are concave and have a smooth texture. The nuchal caputegulae are small and bean-shaped and are oriented anteromedially-posterolaterally on each side of the midline of the skull (Fig. 2A). The medial nuchal caputegulae are smaller than the lateral nuchal caputegulae.

The dorsally-keeled squamosal horns are large and deep compared to those of many other ankylosaurids (Fig. 2E). The tip of the right squamosal horn curves anteriorly; the tip of the left squamosal horn is broken (Fig. 2A, B). The squamosal horn has a sharp dorsoanterior keel, which is not aligned with the keels of the supraorbital caputegulae. The keels of the squamosal horns and those of the supraorbitals are oriented en echelon with respect to the midline of the skull (Fig. 2A). The right squamosal horn measures 10.2 cm from the base to the tip along the ventral surface. An erosional remnant of the left quadratojugal is present and extends lateroventrally from the left side of the skull; the quadratojugal horns are not preserved. Postocular caputegulae may be present, but the bone surface is eroded, so these are difficult to discern (Fig. 2E).

### Palatal Region

The ventral surface of the premaxilla forms part of the secondary palate (Fig. 2B). The surface of the premaxillary secondary palate is concave. Two circular foramina are preserved near the anterior edge of the left premaxilla, although the medial foramen is damaged. Corresponding foramina were probably present on the right premaxilla, but these are obscured by fractures on the ventral surface. A median sagittal slit divides the two premaxillae anteriorly. The posterior halves of the premaxillae are fractured on both sides, and there is a semi-circular depression on the ventral surface on the left side probably due to taphonomic compression. The posterior parts of the premaxillae contact the vomers at the midline. The distance between the posteriormost extent of the premaxillary slit to the anteriormost projection of the vomer is 3.5 cm.

The right tooth row is broken at the posterior end, so its total length cannot be measured. There are 18 tooth positions, and there are four poorly preserved teeth (1st, 2nd, 3rd and 5th), and the base of another (9^th^) preserved. The surfaces of all of the teeth are damaged and individual denticles are not visible. All of the alveoli are damaged on the right maxilla. The left maxilla is weathered on the lateral surface. The length of the left maxillary tooth row is 11.9 cm. The buccal emargination is marked by a tear-drop shaped erosional pit lying midway along the surface. The left maxillary tooth row is broken at the anterior end, and only 16 tooth positions are preserved. The posterior end is damaged. There is a partial tooth at what appears to be position 6, and there is a nearly complete tooth at position 9. The latter tooth has a vertical fracture that splits the tooth in half through its apex. The alveoli are damaged throughout the maxillary tooth row, particularly at the posterior end. The medial borders of both the right and left maxillae form the lateral walls of the paired choanae.

The narrow, sheet-like vomer is preserved along its ventral anterior surface for a distance of 6.0 cm. The anterior extension of the vomer joins the premaxillae at their midline. The vomer is largely missing posteriorly, and only fragments of the vomer have been preserved in situ to allow the reconstruction of this element.

The palatines form a triangular surface on each side of the posterior end of the vomer. The extent of the palatines cannot be determined due to breakage of the vomer medially and of the pterygoids posteriorly. The ventral surface of each palatine forms a single triangular fossa. Although damaged, the palatines lack palatal apertures.

Both ectopterygoids are preserved. The left ectopterygoid is bordered anteriorly by a fenestra measuring 1.0 cm at its widest mediolateral extent. The right ectopterygoid is broken medially and along the anterior part where it contacts the right maxilla. The posterior border of both ectopterygoids forms the anteriormost parts of both post-temporal fenestrae. Only the traces of the pterygoids are visible.

### Occipital/Basicranial Region

The basicranium is composed of the basioccipital posteriorly and the basisphenoid anteriorly. They are separated by a suture that is visible in both left and right lateral views. The suture is oriented in a posterodorsal to anteroventral direction. The basicranium length is 9.4 cm (Fig. 3). The cranial foramina are best preserved on the right side.

The basioccipital has a reniform condyle. The condyle was originally found broken off of the basioccipital, but it has since been rejoined to the skull. Consequently, the present orientation of the condyle may not reflect the precise life position. The maximum width of the condyle is 5.4 cm. The articular surface of the condyle is smooth and surrounded by a rim. The exoccipitals do not contribute to the occipital condyle. In ventral view, the basioccipital has three prominent fossae, flanked by four well-developed walls (Fig. 3B). The medial fossa bears the basioccipital foramen, located at the posterior margin of the fossa. Anteriorly, the basioccipital joins the basisphenoid. A prominent suture between the two elements occurs both ventrally and laterally on each side.

The basisphenoid contacts the midline of the pterygoids anteriorly and the basioccipital posteriorly (Fig. 2B). It has an isosceles triangular shape ventrally, with an anterior apex. In right lateral view the suture between the basisphenoid and the basioccipital is oriented posterodorsally-anteroventrally and extends posterior to three cranial foramina (III, VII and the foramen ovale) on the right side of the basicranium (Fig. 3B). In addition to these cranial foramina, cranial foramina V, IX-X-XI, and XII are also visible. The foramina of the left side of the basicranium are difficult to determine due to poor preservation. The pattern of cranial foramina appears to be largely consistent with those of other ankylosaurids (e.g. [39], [40]), but breaks and suboptimal preservation in this region of the skull make more detailed comparisons difficult.

The exoccipitals are complete but are fractured along their dorsal and distal ends. The distal ends are distinguished by blunt, ventrally directed processes, anterior to which parts of the both the left and right quadrates are fused. The maximum width between the distal ends of the left and right paroccipital processes is 19.0 cm. The supraoccipital lies directly dorsal to the foramen magnum and is fused to the left and right paroccipital processes.

Both the left and right quadrates are largely missing except for the fragmentary portions fused to the distal ends of the left and right paroccipital processes, and to the medial portions of both the quadrate condyles (Fig. 2B, D). The quadratojugals are also missing except for a remnant of the left quadratojugal.

### First Cervical Half Ring

The first cervical half ring (Fig. 4A) preserves the medial part, along with the left and right medial osteoderms, left lateral osteoderm, and the medial basal part of the right lateral osteoderm, coossified to the underlying bony band. The anterior edge of the cervical half ring is largely eroded and missing on the left side, with only the posterior edge preserved. All osteoderms of *Ziapelta* (including cervical and post-cervical) share a common external surface texture characterized by dense, uniform pitting and sparse to no neurovascular grooves. Three of the osteoderms have tall, well-developed keels, although the keels are damaged in places. The bases of the osteoderms are sub-oval in shape. The right medial osteoderm measures 15.5 cm long by 10.4 cm wide and, although the keel is damaged, was at least 9.1 cm tall. The left medial osteoderm has similar proportions, at 15.3 cm long, 7.3 cm wide and 9.5 cm tall. The left lateral osteoderm measures more than 14.6 cm long, 5.8 cm wide, and 10.9 cm tall. Smaller interstitial osteoderms are present between the left and right medial osteoderms. They are distributed irregularly between the basal edges of the left medial and lateral osteoderms. A fractured conical interstitial osteoderm is located along the midline at the anterior edge of the cervical half ring. A partial isolated osteoderm was reconstructed from fragments found near the right side. This appears to be the left distal osteoderm from the first cervical half ring.

### Second Cervical Half Ring

A broken ribbon of bone lies adjacent to the ventral surface of the left medial osteoderm of the first cervical half ring, and extends toward the midline (Fig. 4A). This structure is separated by a wedge of matrix between the osteoderm fused on this element and the ventral side of the left lateral osteoderm of the first cervical half ring. We interpret this structure to be the anterior portion of the left lateral side of the second cervical half ring, which must be offset posteriorly. The inner surface of the half ring is broken and fractured. Two osteoderms are fused to this element, one lying adjacent to the left medial osteoderm of the first cervical half ring and the other near the opposite end of the second cervical half ring. Both these osteoderms are poorly preserved, but each has a discernible outline. They both have an apex; the one lying adjacent to the left lateral osteoderm is directed laterally. This osteoderm measures approximately 9 cm apicobasally. The other osteoderm has an oval base, and the apex is located more toward the midline. It measures 8.6 cm long and 4.8 cm wide. The height of the apex is 2.4 cm. Both osteoderms are situated along the anterior edge of the second cervical half ring. The medial osteoderm has an oval base and a straight anteroposterior keel; although the top of the keel is broken, it does not appear that it had a prominent apex. The lateral osteoderm also has a narrower oval base compared to the medial osteoderm, and has a sigmoidal longitudinal keel. The distal osteoderm has the narrowest base and is dorsoventrally compressed. The keel has a central apex, giving this osteoderm a triangular outline in dorsal view. The dorsal surface of the distal osteoderm is slightly convex. The keels of all osteoderms are directed anteromedially-posterolaterally, with the distal edges of the keels curving dorsomedially. The basal bony band is wider between the paired medial osteoderms than it is between the medial and lateral or lateral and distal osteoderms. Interstitial osteoderms (sensu Arbour and Currie [14]) are present between the medial osteoderms, but between the medial and lateral, or lateral and distal osteoderms.

### Postcervical Osteoderms

Two isolated osteoderms were found lying adjacent to the cervical half rings (Fig. 4A), one between the right side of the skull and anterior border of the first cervical half ring. We identify this as a displaced pectoral osteoderm. The other osteoderm is incomplete but had a relatively large base. This osteoderm also has a well-developed sigmoidal keel and may represent a left lateral osteoderm from the second cervical half ring. This osteoderm is 16.9 cm long with a maximum keel height of 10.3 cm.

A single isolated osteoderm was found posterior and adjacent to the basal surface of the posterior border of the first cervical half ring (Fig. 4A). It is 11.8 cm long and 6.8 cm wide. The keel rises 5 cm to an apex and declines in height 7 cm anteroposteriorly. This osteoderm is compressed and has a sigmoidal keel, which creates a concave dorsal surface on the osteoderm. It is further distinguished by a prominent groove that encircles the entire osteoderm, and is situated about 1 cm from the periphery. We interpret this as a right thoracic osteoderm that was displaced prior to burial.

Lastly, a number of smaller, sub-circular osteoderms were recovered in association with the first and second cervical half rings. Most are relatively complete; three are conical, whereas a few are flat. All have a pitted surface texture. The smallest of these osteoderms measures 2 cm across the width of its base, and the largest measures approximately 4 cm. These may be loose interstitial osteoderms from the cervical half rings or may be from a more posterior body region.

### Referred specimen NMMNH P-66930

An isolated complete first cervical half ring, from the upper part of the Hunter Wash Member of the Kirtland Formation, is referable to *Ziapelta sanjuanensis* based on the presence of interstitial osteoderms and the morphology of the major osteoderms coossified to the band (Fig. 4C). This cervical half ring shares several of the unique features found in the holotype of *Ziapelta*, including proportionately tall and narrow osteoderms compared to those of *Anodontosaurus*, *Euoplocephalus* and *Scolosaurus*, interstitial osteoderms like in *Anodontosaurus* but unlike in *Euoplocephalus*, and a distal osteoderm that does not envelop the terminus of the band as completely as does the distal osteoderm in *Anodontosaurus*, *Euoplocephalus*, and *Scolosaurus*.

## Discussion

### Comparisons with other ankylosaurids

Three ankylosaurids have been recovered from the Kirtland Formation: *Nodocephalosaurus* and *Ziapelta* from the De-na-zin Member, and *Ahshislepelta* from the stratigraphically lower Hunter Wash Member. *Ahshislepelta* and *Ziapelta* cannot be directly compared because there little overlapping material – *Ahshislepelta* is known only from osteoderms and axial and appendicular material, whereas *Ziapelta* is known only from a skull and cervical half rings. Although their external surface textures are similar, consisting of uniform pitting with a sparse pattern of neurovascular grooves, the osteoderms of *Ahshislepelta* are characteristically smoother [11] than either *Nodocephalosaurus* [12] or *Ziapelta*.

**Figure 5:**
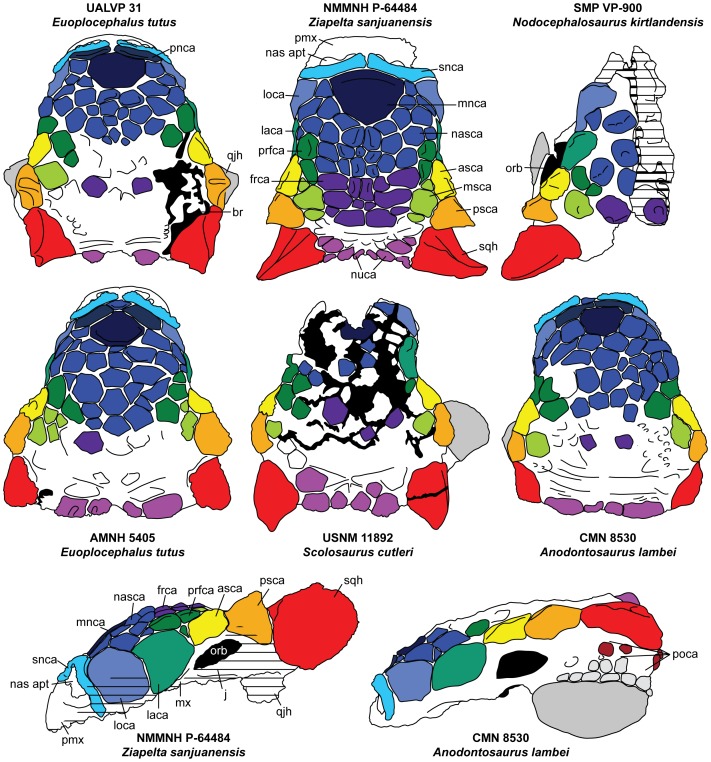
Skull of *Ziapelta sanjuanensis* compared to those of other North American ankylosaurids. Interpretive diagrams scaled to approximate same cranial length (from the anterior edge of the premaxilla to the posterior edge of the nuchal crest) for comparative purposes. Abbreviations: asca, anterior supraorbital caputegulum; br, break/plaster; frca, frontal caputegulum; j, jugal; laca, lacrimal caputegulum; loca, loreal caputegulum; mnca, median nasal caputegulum; msca, medial supraorbital caputegulum; mx, maxilla; nas apt, nasal aperture; nasca, nasal caputegulum; nuca, nuchal caputegulum; orb, orbit; pmx, premaxilla; pnca; postnarial caputegulum; poca, postorbital caputegulum; prfca, prefrontal caputegulum; psca, posterior supraorbital caputegulum; qjh, quadratojugal horn; snca, supranarial caputegulum; sqh, squamosal horn.

Numerous features differentiate *Ziapelta* from *Nodocephalosaurus*, the other ankylosaurid known from the De-na-zin Member of the Kirtland Formation. The holotype of *Nodocephalosaurus kirtlandensis* is a laterally crushed, asymmetric, partial skull [6]. The frontonasal caputegulae of *Nodocephalosaurus* have circular bases, whereas these caputegulae on *Ziapelta* have square, rectangular, or hexagonal bases (Fig. 5). The frontonasal caputegulae in *Nodocephalosaurus* are conical, and none are flat. In *Ziapelta*, these caputegulae can be convex, but many are flat or even concave. The grooves between the caputegulae are more prominent in *Ziapelta* than in *Nodocephalosaurus*, which makes them more distinct. These differences are unlikely to represent ontogenetic changes in a single taxon. In *Pinacosaurus grangeri* Gilmore, 1933 [41], the extent of the frontonasal ornamentation changes from juveniles [42], [43], to adults (AMNH 6526), but the form of the ornamentation does not appear to change substantially. No small juvenile skulls are yet known for *Anodontosaurus* or *Euoplocephalus*, but in the relatively large sample sizes for these taxa [14], which probably include at least some differences in ontogenetic stage, the basal shape of the frontonasal caputegulae does not include the range of variation represented by the holotypes of *Nodocephalosaurus* and *Ziapelta*.

In *Nodocephalosaurus*, the supraorbital caputegulae have distinct peaks, but these are less sharp than the supraorbital caputegulae in *Ziapelta*. *Nodocephalosaurus* has a prominent, bulbous loreal caputegulum (maxillary osteoderm sensu Sullivan [6]) that extends onto the dorsal surface of the skull. This caputegulum in *Ziapelta* is anteroposteriorly broader, and is less convex on its lateral surface and flat on its dorsal surface. In *Nodocephalosaurus*, the squamosal horns are curved ventrally, but in *Ziapelta* the squamosal horns are curved anteriorly. Also, the squamosal horns of *Ziapelta* have a sharper, more prominent dorsal keel compared to those of *Nodocephalosaurus*.

The cranial caputegulum pattern of *Ziapelta* is most similar to that of *Ankylosaurus*, *Anodontosaurus*, *Dyoplosaurus*, *Euoplocephalus*, and *Scolosaurus* from Montana and Alberta ([14]: Fig. 5). Like these taxa, *Ziapelta* has flat, irregularly shaped cranial caputegulae separated by distinct grooves. *Tsagantegia longicranialis* Tumanova, 1993 [44], from the Bayanshiree Formation of Mongolia, also has flat cranial caputegulae, but the skull differs in many ways from that of *Ziapelta*; for example, the skull of *Tsagantegia* is longer than wide, the squamosal horns are shorter and rounder, and the cranial caputegulae are larger and more rectangular. The presence of convex frontonasal caputegulae distinguishes *Ziapelta* from *Ankylosaurus*, *Anodontosaurus*, *Euoplocephalus*, and *Scolosaurus*, and a mixture of convex, flat, and concave frontonasal caputegulae is unknown in any other ankylosaurid. The arrangement of frontonasal caputegulae in *Ziapelta* is more bilaterally symmetrical than in any other North American ankylosaurid taxon.

*Ankylosaurus* differs from all other ankylosaurids by having ventrally-facing narial openings. In *Ziapelta*, the narial openings face anteriorly, and are visible in dorsal view. In many specimens of *Euoplocephalus*, the narial openings face anterolaterally, and are obscured in dorsal view by the supranarial caputegulae. *Euoplocephalus* specimens with narial openings visible in dorsal view tend to be dorsoventrally crushed, like AMNH 5403. Although the holotype skull of *Ziapelta sanjuanensis* does not appear to be greatly crushed, it is possible that some dorsoventral compaction has occurred.

The supranarial caputegulae of *Ziapelta* are similar in shape to those of *Euoplocephalus* and *Anodontosaurus*. In many *Euoplocephalus* skulls (e.g. AMNH 5337, AMNH 5405), the supranarial caputegulae are rugose and pitted and have an irregular surface texture, which differs from the texture of the other cranial caputegulae. In contrast, the supranarial caputegulae of *Ziapelta* are smooth, having a similar texture compared to the other cranial caputegulae.

*Ziapelta* shares with *Ankylosaurus*, *Anodontosaurus*, and *Euoplocephalus* a large median nasal caputegulum, a feature that is absent in the Asian ankylosaurids. However, in *Ankylosaurus*, *Euoplocephalus* and *Anodontosaurus*, the median nasal caputegulum is hexagonal, not triangular (Fig. 5). The median nasal caputegulum occupies a greater proportion of the snout width in *Ziapelta* compared to *Euoplocephalus* and *Anodontosaurus* (50% in *Ziapelta* versus about 40% in *Euoplocephalus* and *Anodontosaurus*). In *Euoplocephalus* and *Anodontosaurus*, there is always a pair of long, horizontally-oriented caputegulae (postnarial caputegulae) between the supranarial caputegulae and median nasal caputegulum, which are absent in *Ziapelta*.

The two supraorbital caputegulae form a continuous lateral edge in dorsal view in *Ankylosaurus*, *Anodontosaurus*, *Euoplocephalus*, *Dyoplosaurus*, and *Scolosaurus*. In contrast, the supraorbital caputegulae of *Ziapelta* each have distinct peaks. Supraorbital caputegulae with distinct peaks are present in the Asian ankylosaurids *Pinacosaurus* and *Tarchia kielanae* Maryańska, 1977 [39] (see also [32]).

The taxonomic significance of squamosal horn shape in ankylosaurids has been controversial [6], [45]–[47]. Squamosal horn shape can vary within *Euoplocephalus* [14]. In dorsal view, squamosal horns can be triangular and pointed (or with a pit at the apex), as in UALVP 31, can be blunt triangles (e.g., AMNH 5405, ROM 1930), or can be nearly indistinguishable from the skull and simply form a rugose posterior corner of the skull (e.g., AMNH 5404, AMNH 5337). These differences could represent ontogenetic changes in *Euoplocephalus*; however, the overall triangular shape is present in all but the largest specimens.

The dorsoventral orientation at which the squamosal horns project from the skull can also vary, but Arbour and Currie [48] suggest that much of this variation can be attributed to dorsoventral compaction during burial and fossilization. Aspects of the squamosal horns that appear to be taxonomically significant include the angle of lateral projection from the skull, curvature of the horn, and overall shape (narrow cylindrical versus pyramidal). *Ziapelta* has unique squamosal horns that are curved anteriorly at the tips, a morphology not observed in any other ankylosaurid. *Scolosaurus* has backswept squamosal horns with ventrally curved tips (e.g. USNM 11892; [14]), and *Nodocephalosaurus* may have slightly ventrally curved tips to the squamosal horns, although this may be the result of plastic deformation in the only known skull. In *Anodontosaurus*, *Ankylosaurus*, and *Euoplocephalus*, the squamosal horns are not lateroventrally ventrally-oriented, nor are they curved in the Asian ankylosaurids [32].

The skull of *Ziapelta* is not constricted anterior to the orbits as seen in *Tarchia kielanae* and *Pinacosaurus grangeri* [32]. Although some of the frontonasal caputegulae of *Ziapelta* are bulbous, they differ from the tall, pyramidal, sharp-edged caputegulae of *Tarchia* and *Saichania chulsanensis* Maryańska, 1977 [39]. In lateral view, the antorbital region of the skull roof is convex in *Ziapelta*, which is similar to the condition in *Anodontosaurus*, *Euoplocephalus*, and *Ankylosaurus*, but unlike the condition in the Asian ankylosaurids.

**Figure 6:**
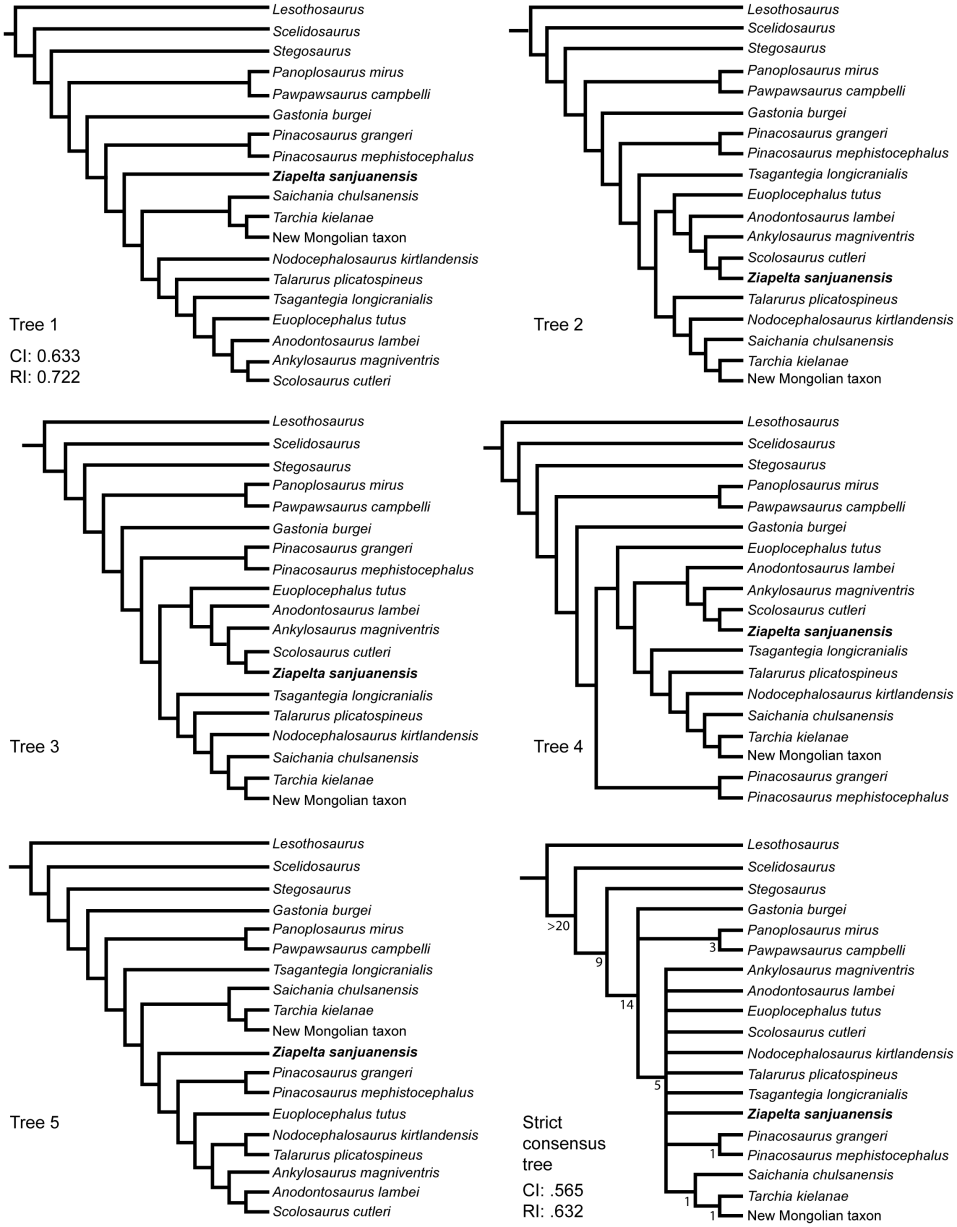
Results of the phylogenetic analyses, showing the relationships of *Ziapelta sanjuanensis* to other ankylosaurids.

*Ziapelta* appears to lack palatal apertures in the palatine bones, which are present in *Anodontosaurus* and *Euoplocephalus* ([14]: fig. 6). The braincase also differs from those of *Anodontosaurus* and *Euoplocephalus*. *Ziapelta* has a deep longitudinal groove along the ventral surface of the basioccipital, and paired fossae on the lateral sides of the basioccipital. These are absent in *Anodontosaurus* and *Euoplocephalus*, and are not likely to be the result of taphonomic compression of NMMNH P-64484, because the fossae are not present even in severely dorsoventrally compressed specimens of *Euoplocephalus* such as AMNH 5403.

The cervical half rings are most similar to those of *Anodontosaurus* and *Euoplocephalus*. The medial osteoderm has a central longitudinal keel, the lateral osteoderm has a sigmoidal keel, and the distal osteoderm is transversely compressed. The cervical half ring osteoderms of *Ziapelta* have more rectangular bases compared to *Ankylosaurus*, *Anodontosaurus*, and *Euoplocephalus*, in which the osteoderms are more elliptical and transversely broader. *Scolosaurus* has low circular medial osteoderms with central apices on the first and second cervical half rings, unlike the condition in *Ziapelta*. An unnamed ankylosaurid (UMNH VP 20202, from the Kaiparowits Formation of Utah, [49]) also has low, indistinct (or absent) medial osteoderms on the first cervical half ring. *Ziapelta* has interstitial osteoderms (two morphotypes) between the major osteoderms of the first cervical half ring, which are absent in *Euoplocephalus* but are present in *Anodontosaurus*. The interstitial osteoderms of *Anodontosaurus* tend to be more circular or square, whereas the interstitial osteoderms of *Ziapelta* are either in the form of a single conical osteoderm, or flat clusters in a sub-trapezoidal shape. The distal osteoderms of the cervical half ring in *Ziapelta* barely overlap the terminus of the band, which differs from the condition in *Ankylosaurus*, *Anodontosaurus*, *Euoplocephalus*, and *Scolosaurus*, in which the distal osteoderms entirely envelop the terminus of the band [14].

At present, *Ziapelta* is not easily compared to any of the ankylosaurid material described from the Kaiparowits Formation of Utah [49]. UMNH VP 19473 includes a cervical half ring, vertebrae, and appendicular elements [49]. Based on the figured material, the cervical half ring may be a second cervical half ring missing the medial osteoderms and preserving the lateral osteoderms. Unfortunately, the poor preservation of the second cervical half ring in *Ziapelta* precludes comparison with UMNH VP 19473. UMNH VP 20202 is a partial skeleton that includes a skull, mandibles, both cervical half rings, vertebrae (including the tail club), and appendicular elements [49]. This specimen has not yet been described or figured in detail, and the skull has not been figured, so comparisons with *Ziapelta* are not possible at present. Loewen et al. [49] also reference an additional undescribed skull from the Kaiparowits Formation that may be distinct from UMNH VP 20202.

### Results of the phylogenetic analysis

Five equally most parsimonious trees were recovered, with a branch-length score of 267 hit 424 times out of 1000, a consistency index of 0.633, and a retention index of 0.722 (Fig. 6). In all trees, *Pinacosaurus* was monophyletic, and a clade containing *Saichania*, *Tarchia*, and a new Mongolian ankylosaurid was recovered. Four of the trees include a clade of North American ankylosaurids (*Ankylosaurus*, *Anodontosaurus*, *Euoplocephalus*, and *Scolosaurus*), three of which also include *Ziapelta*, although the relationships among the North American ankylosaurids are somewhat labile. *Ziapelta* is the sister taxon of *Scolosaurus* in three of the five trees. The relationships of *Tsagantegia*, *Talarurus plicatospineus* Maleev, 1952 [50], and *Nodocephalosaurus* relative to the other taxa in this analysis are most variable, but an analysis of the dataset in TAXEQ3 showed that none of these taxa could be safely removed. Broader taxon sampling in future analyses may help stabilize the position of these ankylosaurids. Nevertheless, *Ziapelta* appears to be most closely allied with the North American taxa *Ankylosaurus*, *Anodontosaurus*, *Euoplocephalus* and *Scolosaurus*, and does not appear to be closely related to the North American species *Nodocephalosaurus*, which is recovered in various Mongolian clades in four of the five trees.

When *Nodocephalosaurus* and *Ziapelta* were constrained as sister-taxa, the minimum tree length was 268, an increase of only one step compared to when *Nodocephalosaurus* and *Ziapelta* are not constrained. In the resulting 8 most parsimonious trees, *Nodocephalosaurus* and *Ziapelta* were never recovered in derived clades containing the North American ankylosaurids.

### Paleobiogeography and biostratigraphy

**Figure 7:**
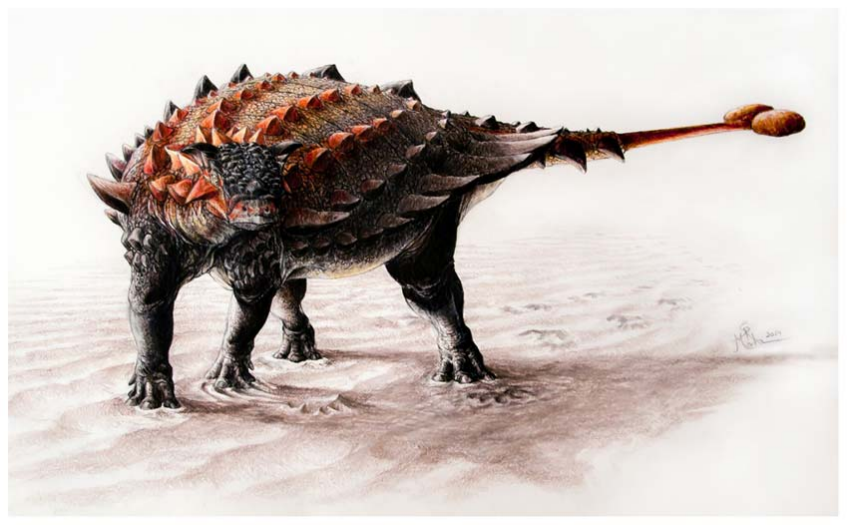
Speculative life restoration of *Ziapelta sanjuanensis*. Illustration by Sydney Mohr.

*Ziapelta* (Fig. 7) provides important new information for understanding the paleobiogeography of ankylosaurid dinosaurs. Sullivan [6] considered *Nodocephalosaurus* to be closely related to the Mongolian ankylosaurids *Saichania* and *Tarchia*, although this was based only on the presence of bulbous caputegulae on the skull, and not the result of a phylogenetic analysis. The presence of an ankylosaurid closely related to Mongolian taxa in the late Campanian of New Mexico in turn suggested some palaeogeographic connection between Asia and western North America at or somewhat before that time [6]. Thompson et al. [51] recovered the North American *Dyoplosaurus acutosquameus* as the sister taxon to the Chinese *Pinacosaurus mephistocephalus* Godefroit, Pereda-Suberbiola, Li, and Dong, 1999 [52] from the Bayan Mandahu Formation (Campanian), Inner Mongolia, People's Republic of China, which at first may seem to provide support for an intercontinental exchange of ankylosaurids. However, Arbour and Currie [14] found the characters uniting these two taxa to represent inaccurate character codings: revising the codings of several characters, including the “doming” of the parietal surface, resulted in a loss of the close relationship between *Dyoplosaurus acutosquameus* and *Pinacosaurus mephistocephalus*. Although *Ziapelta* and *Nodocephalosaurus* occur in the same formation, *Ziapelta* is more closely related to northern North American ankylosaurids than to *Nodocephalosaurus*.

*Ziapelta* shares several features with *Ankylosaurus*, *Anodontosaurus*, *Euoplocephalus*, and *Scolosaurus*, such as the flat, square-to-hexagonal based cranial caputegulae and a convex antorbital region of the skull roof. However, it also has some bulbous, convex cranial caputegulae, which are otherwise known only in *Nodocephalosaurus* and the derived Asian ankylosaurids. Based on the results of the phylogenetic analysis, bulbous cranial caputegulae appear to have evolved independently in *Nodocephalosaurus* and *Ziapelta*. Additional taxon sampling of Early Cretaceous Asian ankylosaurids may help resolve the relationships of *Nodocephalosaurus* and the Campanian-Maastrichtian Asian ankylosaurids, and to address questions about ankylosaurid dispersal between North America and Asia.

Does the presence of *Ziapelta* in the southern portion of Laramidia support current hypotheses of distinct southern and northern North American dinosaur faunas (e.g. [53], [54])? *Ziapelta* is not known from Montana or Alberta, and *Anodontosaurus*, *Euoplocephalus*, *Dyoplosaurus*, and *Scolosaurus* have not been recovered south of Montana [14]. However, identifying northern and southern faunal provinces requires that the representative faunas are coeval. The Dinosaur Park Formation, upper portions of the Judith River and Two Medicine formations, and Kaiparowits Formation overlap in time, but the Kirtland Formation represents a younger time interval [1], [16], [55]. The holotype of *Ziapelta sanjuanensis* was collected from the De-na-zin Member of the Kirtland Formation, approximately 12 m below Ash H, which has recently been recalibrated to 72.98±0.18 Ma by Roberts et al. [16]. In Alberta, the equivalent time is represented by the lower portion of the Drumheller Member of the Horseshoe Canyon Formation [56]. No identifiable ankylosaurids have been recovered from this part of the Horseshoe Canyon Formation (Fig. 1); *Anodontosaurus* is known from a few specimens in the upper part of the Dinosaur Park Formation (∼75 Ma), and from the Horsethief Member (71.5 Ma [56]), Morrin Member, and Tolman Member of the Horseshoe Canyon Formation [14]. *Dyoplosaurus*, *Euoplocephalus*, and *Scolosaurus* occur in older sediments than *Ziapelta*, and *Ankylosaurus* is known from younger sediments. As a result, the presence of *Ziapelta* in the Kirtland Formation, outside of the Kaiparowits-Dinosaur Park- upper Two Medicine taphozone (sensu Roberts et al. [16]), cannot be used to support hypotheses of dinosaur provincialism at this time; additional diagnostic ankylosaurid specimens from the Drumheller Member of the Horseshoe Canyon Formation, or other time-equivalent strata, are needed to clarify the paleobiogeography of Campanian-Maastrichtian ankylosaurids from Laramidia.

Since 1999, *Nodocephalosaurus* has been considered the only ankylosaurid from the De-na-zin Member of the Kirtland Formation. The small *Ahshislepelta minor* is known only from the holotype from the slightly older Hunter Wash Member [11]. The nodosaurid *Glyptodontopelta* had been considered restricted to the Maastrichtian Naashoibito Member of the Ojo Alamo Formation [18], and a taxon characteristic of the Alamo Wash local fauna [2], [9], [10]. The diversity of ankylosaurids in the San Juan Basin appears to be higher than that of nodosaurids based on the specimens currently known, and all of the named ankylosaurid taxa are characteristic of the Kirtlandian Land Vertebrate Age [1], [55]. *Nodocephalosaurus* may be a taxon unique to the Willow Wash local fauna, and *Ahshislepelta* unique to the older Hunter Wash local fauna.

## Conclusions

*Ziapelta sanjuanensis*, gen. et sp. nov., is a new anklyosaurid based on a well-preserved skull, incomplete first and second cervical half rings and isolated body and cervical half ring osteoderms. It was found low in the De-na-zin Member of the Kirtland Formation, stratigraphically below, and close to, the holotype locality of *Nodocephalosaurus kirtlandensis*, yet it appears to be more closely related to *Scolosaurus* from the chronostratigraphically older Dinosaur Park Formation of Alberta, than to *Nodocephalosaurus*, based on three of the five most parsimonious trees. Additional character and taxon sampling are needed in order to further resolve ankylosaurid relationships, but our analysis suggests that the two ankylosaurids from the De-na-zin Member of the Kirtland Formation are not most closely related to each other.

## Supporting Information

File S1 **Specimens examined and character definition statements.**
(DOCX)

File S2 **Character-taxon matrix.**
(NEX)
