# Supplementary material for: A New Ankylosaurid Dinosaur from the Upper Cretaceous (Kirtlandian) of New Mexico with Implications for Ankylosaurid Diversity in the Upper Cretaceous of Western North America
Source: PLoS One. 2014 Sep 24;9(9):e108804. doi: 10.1371/journal.pone.0108804 (PMC4177562; doi:10.1371/journal.pone.0108804)
Supplement: File S1 — Specimens examined and character definition statements. (DOCX) [file pone.0108804.s001.docx]

**Supporting Information File S1: Specimens Examined and Character Statements**

**Part 1: Specimens Examined**

**Institutional Abbreviations: AMNH** – American Museum of Natural History, New York, New York, USA; **CMN** – Canadian Museum of Nature, Ottawa, Ontario, Canada; **DMNH** – Denver Museum of Nature and Science, Denver, Colorado, USA; **FPDM-V** – Fukui Prefectural Dinosaur Museum (Vertebrate Collection), Katsuyama, Fukui Prefecture, Japan; **INBR** – Victor Valley Museum, Apple Valley, California, USA; **IVPP** – Institute for Vertebrate Paleontology and Paleoanthropology, Beijing, China; **MACN Pv** – Colección nacional de Paleontología de Vertebrados del Museo Argentino de Ciencias Naturales “Bernardino Rivadavia”, Buenos Aires, Argentina; **MPC** – Paleontological Center, Mongolian Academy of Sciences, Ulaanbaatar, Mongolia (MPC KID refers to Korea-Mongolia Dinosaur Project field numbers); **MOR** – Museum of the Rockies, Bozeman, Montana, USA; **NHMUK** – Natural History Museum, London, United Kingdom; **NMMNH** – New Mexico Museum of Natural History and Science, Albuquerque, New Mexico, USA; **PIN** – Palaeontological Institute, Russian Academy of Sciences, Moscow, Russia; **ROM** – Royal Ontario Museum, Toronto, Ontario, Canada; **RSM** – Royal Saskatchewan Museum, Regina, Saskatchewan, Canada; **SMP** – State Museum of Pennsylvania, Harrisburg, Pennsylvania, USA**; TMP** – Royal Tyrrell Museum of Paleontology, Drumheller, Alberta, Canada; **UALVP** – University of Alberta Laboratory for Vertebrate Paleontology, Edmonton, Alberta, Canada; **USNM** – Smithsonian Museum of Natural History, Washington, DC, USA; **ZPAL** – Zaklad Paleobiologii, Polish Academy of Sciences, Warsaw, Poland.

| *Ahshislepelta minor* | SMP VP-1930 (holotype: associated incomplete postcranial skeleton, including both scapulocoracoids, left humerus, proximal portion of left radius, vertebral fragments, osteoderms, and unidentifiable fragments). |
| --- | --- |
| *Ankylosaurus magniventris* | AMNH 5895 (holotype: three caudal vertebrae, portions of both cervical half rings).  AMNH 5214, complete skull, both mandibles, seven caudal vertebrae including tail club, on display; cast of skull UALVP 54722. |
| *Anodontosaurus lambei* | CMN 8530 (holotype: skull, lower jaws, caudal vertebra, ischium, pedal phalanx, and osteoderms including first cervical half ring).  AMNH 5216 (tail club), AMNH 5223 (skull), AMNH 5245 (caudosacral and caudal vertebra, pelvis, tail club).  NHMUK R4947 (skull).  ROM 832 (fragmentary skull).  TMP 1982.9.3 (two posterior dorsals with coossifed ribs, partial pelvis, right femur, osteoderms including cervical half ring fragments), TMP 1994.168.1 (tail club), TMP 1996.75.01 (partial skull, cervical vertebra, partial ?first cervical half ring, ?second cervical half ring), TMP 1997.59.1 (skull), TMP 1997.132.01 (skull, three dorsal vertebrae, ribs, ?scapula, left humerus, ulna, radius, tibia, first and possibly second cervical half rings).  USNM 10753 (tail club). |
| *Dyoplosaurus acutosquameus* | ROM 784 (holotype: fragmentary skull, complete caudal series of vertebrae including tail club, ribs, pelvis, hindlimb including pes, osteoderms *in situ*).  UALVP 47273 (partial tail club). |
| *"Dyoplosaurus giganteus"* | PIN 551/29 (holotype: caudal vertebrae, metatarsals, phalanges, osteoderms including tail club knob) |
| *Euoplocephalus tutus* | CMN 210 (holotype, fragmentary skull roof and partial first cervical half ring).  AMNH 5337 (skull, left mandible, one cervical vertebra, eleven dorsal vertebrae, humeri, scapulocoracoid, pelvis, osteoderms including first cervical half ring), AMNH 5403 (skull, both mandibles including predentary, four cervicals including axis, scapula, forelimbs, first and second cervical half rings, partial tail club knob), AMNH 5404 (skull, five caudals, ribs, right humerus, ischium, right femur, tibia, fibula, osteoderms, first cervical half ring), AMNH 5405 (skull, right mandible including predentary, handle vertebrae, humerus, ulna, osteoderms, first cervical half ring, tail club knob), AMNH 5406 (three dorsal vertebrae, ribs, scapulae, right humerus, ulna, radius, phalanges, osteoderms including first and second cervical half rings).  CMN 842 (first cervical half ring), CMN 8876 (skull).  ROM 1930 (skull, three dorsal vertebrae, two ?sacral vertebrae, twelve free caudals, transitional caudal, fragmentary right scapula, right humerus, osteoderms including *in situ* osteoderms and skin impressions on caudal vertebrae).  TMP 1979.14.74 (partial skull).  UALVP 31 (skull, right mandible, ribs, sacrum, scapula, humeri, right ilium, right ischium, right femur, tibia, pedal elements, osteoderms including first and second cervical half rings), UALVP 47977 (partial skull roof). |
| *Gastonia burgei* | CEUM 10293 (skull), CEUM cast pelvic shield, CEUM (holotype skull), cast of holotype skull UALVP 54755.  Bonebed material, mostly caudals, at DMNH |
| *Glyptodontopelta mimus* | USNM 8610 (holotype: portion of pelvic shield, isolated thoracic and pelvic osteoderms, and fragments from cervical half rings), NMMNH P-14266 (osteoderms), NMMNH P-25063 (cervical osteoderms), NMMNH P-27420 (osteoderm), NMMNH P-27450 (cervical osteoderm), NMMNH P-27849 (osteoderms), SMP VP-1147 (osteoderms), SMP VP-1319 (osteoderm), SMP VP-1580 (71 nearly complete osteoderms, numerous fragmentary osteoderms, and indeterminate postcranial fragments), SMP VP-1640 (osteoderms), SMP VP-1731 (osteoderm), SMP VP-1825 (osteoderm), SMP VP-1831 (osteoderm), SMP VP-1832 (osteoderm), SMP VP-1863 (osteoderm), SMP VP-2067 (osteoderms and indeterminate postcranial fragments), SMP VP-2077 (osteoderms), USNM 8611 (several osteoderm fragments) |
| *Nodocephalosaurus kirtlandensis* | SMP VP-900 (holotype: partial skull), SMP VP-1957 (fragment of skull roof with two caputegulae, possibly representing the frontal region), NMMNH P-27405 (osteoderms) |
| *Panoplosaurus mirus* | CMN 2769 (holotype: skull, sacrum, pes, osteoderms) |
| *Pawpawsaurus campbelli* | Cast of holotype, UALVP 54698 (skull) |
| *Pinacosaurus grangeri* | AMNH 6523 (holotype: skull and mandibles)  MPC 100/1305 (complete skeleton with *in situ* osteoderms but lacking skull and cervical half rings, on display at MPC)  PIN 614 (complete skeleton with osteoderms but lacking skull, on display at the Orlov Museum of Paleontology (Russian Academy of Sciences). PIN 3780/3 (skull).  ZPAL MgD II/1 (skull, mandibles, predentary, cervicals, dorsal, caudals, ribs, scapula, coracoids, humerus, radius, ulna, ilium, both femora, tibia, fibula, cervical half ring fragments). ZPAL MgD II/9 (caudals, tail club handle, pelvis, manus, femur, tibia, pes), ZPAL MgD II/31 (tail club handle);  From the Alag Teeg Bonebed: MPC 100/1307, pedal elements; MPC 100/1308 tibiae, pedal elements; MPC 100/1309, pedal elements; MPC 100/1310, left forelimb; MPC 100/1311, hindlimb and pedal elements; MPC 100/1312, pedal elements; MPC 100/1313, pedal elements; MPC 100/1315, manual elements from two individuals; MPC 100/1316 , tibiae, fibulae, pedal elements; MPC 100/1317, manual elements; MPC 100/1318, manual elements; MPC 100/1319, left pes; MPC 100/1320, tibia, fibula, right pes; MPC 100/1321, skull and postcranial elements; MPC 100/1322, quadrates, coracoid, ulna, ischium, three femora of two individuals; MPC 100/1323, right ulna, manus, both pedes; MPC 100/1324, vertebrae, coracoid, osteoderms; MPC 100/1325, right manus; MPC 100/1326, forelimb, manus, associated caudal vertebrae; MPC 100/1327, left tibia and pes; MPC 100/1328, left pes; MPC 100/1329, vertebrae, manual and pedal elements; MPC 100/1330, cervical half ring fragments, vertebrae, manus; MPC 100/1331, complete right pes; MPC 100/1332, scapula, coracoid, ribs; MPC 100/1333, ilia, humeri, radius, ulna, both manus, pes (large individual); MPC 100/1334, tibia, fibula, pedal elements; MPC 100/1335, skull, forelimbs, hindlimb; MPC 100/1335, osteoderms; MPC 100/1337, right manus; MPC 100/1338, manual elements; MPC 100/1339, right ulna, radius, manus, tibiae, fibulae, both pedes; MPC 100/1340, radius, ulna, manus; MPC 100/1341, radius, ulna, manus; MPC 100/1342, tibia, fibula, pes; MPC 100/1343, hindlimb, both pedes; MPC 100/1344, skull, cervical vertebrae, cervical half ring, articulated dorsal vertebrae, scapula, humerus, both femora, tail; MPC 100/1345, cervical half ring, rib; MPC 100/1346, coracoid, humerus; MPC 100/1347, frontal. Additional undescribed Alag Teeg material in the Hayashibara Museum collections, and at the PIN.  From Bayan Mandahu: IVPP V16853, skull and cervical half rings (Bayan Mandahu, locality 100); IVPP V16283, partial skull (Bayan Mandahu, locality 100), IVPP V16854, nearly complete skeleton with skull (Bayan Mandahu, locality 101); IVPP V16346, partial skull (Bayan Mandahu, locality 106); IVPP V16855, skull and skeleton (Bayan Mandahu, unknown locality). |
| *Saichania chulsanensis* | Cast of holotype skull mounted with MPC 100/1305.  Cast of *in situ* holotype skeleton at ZPAL Museum of Evolution.  PIN 3142/250 (complete skull, both mandibles, and predentary; some osteoderms on display) |
| *Scelidosaurus harrisonii* | NHMUK R1111 (cast of complete skeleton, axis, dorsal, two caudals with osteoderms); specimens on display at the Bristol City Museum |
| *Scolosaurus cutleri* | NHMUK R5161 (holotype: nearly complete skeleton with *in situ* osteoderms and skin impressions, lacking skull, distal half of tail, right forelimb, and right hindlimb).  MOR 433 (partial skull, both humeri, free caudal vertebra, and osteoderms).  TMP 2001.42.19 (skull, partial first cervical half ring, dorsals, sacrals, caudals including complete tail club, left humerus, left scapula, right femur, right and left tibiae, osteoderms)  USNM 7943 (partial first cervical half ring), USNM 11892 (skull). |
| *Talarurus plicatospineus* | PIN 557 (holotype: partial skull (PIN 557-3) and postcranial skeleton; mounted composite specimen on display at PIN).  Undescribed material collected by the Korea-Mongolia Joint International Dinosaur Project, from Bayn Shiree, includes: MPC KID 154 (dorsal vertebra neural arch), MPC KID 167 (dorsal vertebra, partial cervical half ring), MPC KID 185 (partial coracoid), MPC KID 186 (quadrate, quadratojugal horn), MPC KID 187 (free caudal centrum with fused haemal arch, cervical half ring fragments, possible tail club knob fragments), MPC KID 166 (skull). Undescribed material collected by the Korea-Mongolia Joint International Dinosaur Project, from Shine Us Khudag, includes: MPC KID 151 (braincase), KID MPC 155 (seven dorsals, three caudal vertebrae, ribs, ilia and sacrum, both ischia, osteoderms, ossified tendons), MPC KID 162 (bonebed collection - skull roof fragments, quadrate, unidentified cranial fragments, dentary fragment, two caudal vertebrae, pathological rib, tibia with coossified astragalus, distal fibula with coossified calcaneum and distal tarsal, phalanges, cervical ring fragments, osteoderm, indeterminate fragments; some non-ankylosaurian material as well). |
| *Tarchia kielanae* | UALVP 49402, cast of INBR 21004 (holotype of *Minotaurasaurus*; skull and jaws) |
| *Tsagantegia longicranialis* | MPC 700/17 (holotype: skull) |
| *Zaraapelta nomadis* | MPC D100/1388 (skull) |
| *Ziapelta sanjuanensis* | NMMNH P-64484 (nearly complete skull, left side of first cervical half ring, partial second cervical half ring, post-cervical osteoderms), NMMNH P-66930 (complete first cervical half ring) |
| Ankylosauridae indet. Alberta | AMNH 5211 (tail club), AMNH 5266 (juvenile individual with vertebrae, ischium, right hindlimb with pes).  CMN 125 (skull roof fragment), CMN 135 (tail club knob), CMN 268 (fragmentary first cervical ring), CMN 349 (tail club), CMN 2251 (partial tail club knob), CMN 2252 (partial tail club knob), CMN 2253 (partial tail club knob).  MACN Pv 12554 (tail club).  NHMUK R8265 (left quadratojugal horn), NHMUK R36629 (?posterior supraorbital), NHMUK R36630 (quadratojugal horn), NHMUK R36631 (?squamosal horn).  ROM 788 (tail club), ROM 813 (partial skeleton with *in situ* osteoderms, skin impressions), ROM 7761 (tail club knob).  TMP 1967.13.2 (tail club knob fragment), TMP 1967.19.4 (left squamosal horn), TMP 1967.20.20 (right quadratojugal horn), TMP 1979.14.164 (partial skull), TMP 1980.8.284 (supraorbital), TMP 1980.16.1685 (fragmentary right mandible), TMP 1983.36.120 (tail club), TMP 1984.121.33 (partial tail club knob), TMP 1985.36.70 (free caudal vertebra), TMP 1985.36.330 (highly fragmentary skull in numerous pieces), TMP 1988.106.5 (left supraorbital), TMP 1991.36.321 (fragmentary first cervical ring), TMP 1991.36.743 (portion of frontonasal region), TMP 1992.36.334 (free caudal vertebra), TMP 1992.36.421 (right mandible), TMP 1993.36.79 (left squamosal), TMP 1993.36.421 (tail club), TMP 1998.83.1 (skull, cervical half ring: indeterminate because unprepared as of 2012), TMP 1993.66.13 (quadratojugal horn), TMP 1996.12.15 (portion of supraorbital region), TMP 1997.36.313 (right mandible), TMP 1998.93.55 (free caudal vertebra), TMP 1998.93.65 (free caudal vertebra), TMP 2000.57.3 (phalanges, tail club), TMP 2000.57.30 (portion of lacrimal/frontonasal region), TMP 2003.12.166 (fragmentary ?second cervical ring), TMP 2003.12.169 (first cervical ring distal osteoderm), TMP 2003.12.311 (skull, cervical half ring: indeterminate because unprepared as of 2012), TMP 2004.98.06 (mandible), TMP 2005.09.75 (free caudal), TMP 2005.12.43 (free caudal vertebra), TMP 2005.49.178 (portion of frontonasal region), TMP 2007.020.0063 (small quadratojugal horn), TMP 2007.20.80 (free caudal vertebra), TMP 2007.12.52 (second cervical half ring), TMP 2007.20.100 (free caudal vertebra), TMP 2012.005.2 (portion of lacrimal/frontonasal region).  UALVP 16247 (tail club), UALVP 45931 (partial first and second cervical half rings), UALVP 47273 (tail club), UALVP 49314 (anterior supraorbital), UALVP 52875 (partial tail club knob), UALVP 54685 (posterior supraorbital).  Additionally, many isolated osteoderms and teeth from the Dinosaur Park Formation are in the TMP and UALVP collections. |
| Ankylosauridae indet. Mongolia | Baruungoyot Fm.: PIN 3142/251 (tail club on display at PIN, cast at DMNH)  Nemegt Fm.: PIN 5011/87 (first cervical half ring, on display at PIN as *Tarchia*); MPC KID 373 (partial dentary); MPC KID 515 (dorsal vertebrae, pedal phalanx, and osteoderms); MPC KID 538 (partial tail club handle); KID 586 (humerus); MPC KID 589 (cervical half ring fragment); MPC KID 591 (free caudal vertebra and osteoderms); MPC KID 630 (humerus); MPC KID 636 (free caudals, handle caudal, osteoderms); MPC KID 637 (free caudal, osteoderms); numerous isolated osteoderms or clusters of osteoderms from MPC KID expeditions; ZPAL MgD I/42 (tail club); ZPAL MgD I/43 (tail club, housed at MPC) |
| Ankylosauridae indet. Montana | AMNH 5470 (partial sacrum), AMNH 20870 (handle vertebrae).  MOR 363 (braincase, both quadratojugal horns, and skull roof fragments).  USNM 16747 (handle vertebrae). |
| Ankylosauridae indet. New Mexico | NMMNH P-22654 (osteoderms), SMP VP-736 (caudal vertebra), SMP VP-975 (dorsal vertebra), SMP VP-1632 (tail club knob osteoderm), SMP VP-1646 (partial tail club knob), SMP VP-1743 (caudal vertebra), SMP VP-1870 (osteoderm), SMP VP-2075 (osteoderm), SMP VP-2074 (partial tail club knob), USNM 8360 (humerus) |

**Part 2: Character Statements and Codings**

**General notes:** Character statements and codings follow those in Arbour et al. (in press), which removes several characters from Thompson et al.'s (2012) matrix, and which uses the updated character codings presented in Arbour and Currie (2013). Some additional characters were removed from the character list in Arbour et al. (in press):

- Character 18, shape of the ventral margin of premaxillary tomium in lateral view; this region is prone to breakage because the bones are so thin, making it difficult to determine the appropriate character state
- Character 63, ornamentation present or absent; this character was subsumed into characters discussing the morphology of cranial ornamentation
- Character 69, squamosal horn present or absent; this character was subsumed into characters discussing the morphology of the squamosal horn
- Character 70, quadratojugal horn present or absent; this character was subsumed into characters discussing the morphology of quadratojugal horn
- Character 75, mandibular caputegulum present or absent; this character was subsumed into characters discussing the morphology of the caputegulum
- Character 139, sacral shield of fused osteoderms present or absent; this character was subsumed into characters discussing the morphology of the sacral shield
- Character 141, tail club knob present or absent; this character was subsumed into characters discussing the morphology of the tail club knob

**Characters:**

1. Antorbital fenestra: present (0); absent (1). (Sereno 1999: 8, Thompson et al. 2012: 1, Arbour et al. in press: 1)**.**
2. Lateral temporal fenestra, visible in lateral view: visible (0); not visible (1). (Carpenter et al. 1998: 6 Thompson et al. 2012: 2, Arbour et al. in press: 2).
3. Supratemporal fenestra: open (0); closed (1). (Lee 1996: 2, Thompson et al. 2012: 3, Arbour et al. in press: 3).
4. Skull dimensions, including ornamentation: longer than wide (0); as wide, or wider than long (1). (Carpenter et al. 1998: 1, Thompson et al. 2012: 4, Arbour et al. in press: 4).
5. Width of the posterior margin of the skull (including squamosal horns) relative to the maximum width across the orbits: greater or equal (0); less (1). (Vickaryous et al. 2004: 6, Thompson et al. 2012: 5, Arbour et al. in press: 5).
6. Size of occiput: higher than wide (0); wider than high (1). (Sereno 1999: 99, Thompson et al. 2012: 6, Arbour et al. in press: 6).
7. External nares, defined as the outermost rim of the nasal vestibule, opening faces: laterally (0); anterolaterally (1); anteriorly (2). (Carpenter et al. 1998: 10; Thompson et al. 2012: 7, Arbour et al. in press: 7).
8. External nares, visible in dorsal view: visible (0); hidden (1). (Thompson et al. 2012: 8, Arbour et al. in press: 8)
9. Orbits, angle of orbital axis: <40º (0); >40º (1). (Thompson et al. 2012: 13, Arbour et al. in press: 9).
10. Antorbital region of the dorsal skull surface: flat (0); domed (1). (Sereno 1999: 99; Thompson et al. 2012: 14, Arbour et al. in press: 10).
11. Development of the postocular shelf: not developed (0); completely separating orbit from temporal space (1). (Sereno 1999: 104; Thompson et al. 2012: 15, Arbour et al. in press: 11).
12. Gap between palate and braincase: open (0); closed by a dorsal projection of the pterygoid (1). (Sereno 1999: 61; Thompson et al. 2012: 16, Arbour et al. in press: 12).
13. Dimensions of premaxillary palate: longer than wide (0); wider than long (1). (Vickaryous et al. 2001: 13; Thompson et al. 2012: 18, Arbour et al. in press: 13).
14. Shape of the premaxillary palate: sub-triangular (0); sub-quadrate (1); sub-oval (2). (Sereno 1999: 80; Thompson et al. 2012: 19, Arbour et al. in press: 14).
15. ‘V’ or ‘U’-shaped median indentation of the anterior margin of the premaxilla: absent (0); present (1). (Sereno 1999: 91; Thompson et al. 2012: 20, Arbour et al. in press: 15).
16. Caudoventral extension of premaxillary tomium in lateral view: ends anteriorly to the maxillary teeth (0); obscures anteriormost maxillary teeth (1). (Sereno 1999: 100; Thompson et al. 2012: 21, Arbour et al. in press: 16).
17. Bone bordering anterior margin of internal nares: premaxilla (0) maxilla (1). (Thompson et al. 2012: 22, Arbour et al. in press: 17).
18. Shape of the maxillary tooth row: straight (0); medially convex (1). (Vickaryous et al. 2001: 18; Thompson et al. 2012: 24, Arbour et al. in press: 18).
19. Maxillary tooth row position: lateral margin of skull (0); inset (1). (Lee 1996: 4; Thompson et al. 2012: 25, Arbour et al. in press: 20).
20. Distance between posteriormost extent of maxillary tooth rows relative to the width of the premaxillary beak: wider (0); narrower (1). (Sereno 1999: 102; Thompson et al. 2012: 26, Arbour et al. in press: 21).
21. Anterior and posterior supraorbitals (recognisable by distinct regions of ornamentation above the orbit): absent (0); present (1). (Sereno 1999: 13; Thompson et al. 2012: 29, Arbour et al. in press: 22**)**.
22. Form of supraorbital ornamentation: boss-like, rounded laterally (0); sharp lateral rim, forming a ridge (1). (Vickaryous et al. 2001: 5; Thompson et al. 2012: 30, Arbour et al. in press: 23).
23. Proportions of jugal orbital ramus: depth greater than transverse breadth (0); transverse breadth greater than depth (1). (Sereno 1999: 1; Thompson et al. 2012: 32, Arbour et al. in press: 24).
24. Shape of quadrate in lateral aspect: curved (anteriorly convex, posteriorly concave) (0); straight (1). (Vickaryous et al. 2001: 38; Thompson et al. 2012: 33, Arbour et al. in press: 25).
25. Inclination of quadrate in lateral aspect: near vertical (0); almost 45º rostrolaterally (1). (Lee 1996: 10; Thompson et al. 2012: 34, Arbour et al. in press: 26).
26. Form of the anterior surface of the quadrate: transversely concave (0); not concave (1). (Lee 1996: 12; Thompson et al. 2012: 35, Arbour et al. in press: 27).
27. Ventral projection of the mandibular process of the quadrate in lateral view: projects beyond the quadratojugal ornamentation (0); hidden by quadratojugal ornamentation (1). (Vickaryous et al. 2004 : 40; Thompson et al. 2012: 36, Arbour et al. in press: 28).
28. Form of quadrate mandibular extremity: symmetrical (0); medial condyle larger than lateral condyle (1). (Sereno 1999: 10; Thompson et al. 2012: 37, Arbour et al. in press: 29).
29. Inclination of the articular surface of the quadrate condyle in posterior view: horizontal (0); ventromedially inclined at approximately 45° to horizontal (1). (Sereno 1999: 14; Thompson et al. 2012: 38, Arbour et al. in press: 30).
30. Lateral ramus of the quadrate: present (0); absent (1). (Sereno 1999: 15; Thompson et al. 2012: 39, Arbour et al. in press: 31).
31. Dorsoventral depth of the pterygoid process of the quadrate: deep (0); shallow (1). (Lee 1996: 7; Sereno 1999: 60; Thompson et al. 2012: 40, Arbour et al. in press: 32).
32. Contact between paroccipital process and quadrate: sutural (0); fused (1). (Carpenter et al. 1998: 13; Thompson et al. 2012: 41, Arbour et al. in press: 33).
33. Contact between pterygoids: pterygoids separate caudomedially, forming an interpterygoid vacuity (0); pterygoids joined medially forming a pterygoid shield (1). (Thompson et al. 2012: 42, Arbour et al. in press: 34).
34. Direction of the pterygoid flange: anterolateral (0); anterior/parasagittal (1). (Vickaryous et al. 2001: 29; Thompson et al. 2012: 43, Arbour et al. in press: 35).
35. Contact between basipterygoid processes and pterygoid: sutural (0); fused (1). (Vickaryous et al. 2001: 30; Thompson et al. 2012: 44, Arbour et al. in press: 36).
36. Position of ventral margin of the pterygovomerine keel relative to alveolar ridge: dorsal (0); level (1). (Sereno 1999: 59; Thompson et al. 2012: 45, Arbour et al. in press: 37).
37. Dorsal extent of median vomer lamina: does not meet skull roof (0); meets skull roof (1). (Lee 1996: 14; Thompson et al. 2012: 46, Arbour et al. in press: 38).
38. Pterygoid foramen: absent (0); present (1). (Hill et al. 2003: 21; Thompson et al. 2012: 47, Arbour et al. in press: 39).
39. Position of posterior margin of pterygoid body relative to the anterior margin of the quadrate condyle: anteriorly positioned (0); in transverse alignment (1). (Vickaryous et al. 2004: 28, Thompson et al. 2012: 48, Arbour et al. in press: 40).
40. Caudoventral secondary palate: absent (0); present (1). (Vickaryous et al. 2004:21, Thompson et al. 2012: 49, Arbour et al. in press: 41)
41. Posterior palatal foramen: absent (0); present (1). (Lee 1996: 17; Thompson et al. 2012: 50, Arbour et al. in press: 42).
42. Direction of paroccipital process extension: caudolateral (0); lateral (1). (Carpenter et al. 1998: 11; Vickaryous et al. 2004: 33 ; Thompson et al. 2012: 51, Arbour et al. in press: 43).
43. Bones forming the occipital condyle: basioccipital and exoccipital (0); basioccipital only (1). (Lee 1996: 9; Thompson et al. 2012: 54, Arbour et al. in press: 44).
44. Length of basisphenoid relative to the basioccipital: longer (0); shorter or equal (1). (Sereno 1999: 12; Thompson et al. 2012: 56, Arbour et al. in press: 45).
45. Form of basisphenoidal tuberosities: medially separated rounded rugose stubs (0); continuous transverse rugose ridge (1). (Vickaryous et al. 2001: 32; Thompson et al. 2012: 57, Arbour et al. in press: 46).
46. Size of basipterygoid processes: twice as long as wide or over (0); less than twice as long as wide (1). (Thompson et al. 2012: 58, Arbour et al. in press: 47).
47. Form of the cranial nerve foramina IX-XII: separate foramina (0); single foramen shared with the jugular vein (1). (Thompson et al. 2012: 59, Arbour et al. in press: 48).
48. Direction of occipital condyle: posterior (0); posteroventral (1). (Vickaryous et al. 2004: 36, Thompson et al. 2012: 61, Arbour et al. in press: 49).
49. Direction of the foramen magnum: posterior (0); posteroventral (1). (Vickaryous et al. 2004: 37, Thompson et al. 2012: 62, Arbour et al. in press: 50).
50. Premaxillary teeth: present (0); absent (1). (Sereno 1999: 18; Thompson et al. 2012: 63, Arbour et al. in press: 51).
51. Cingula on maxillary and/or dentary teeth: absent (0); present (1). (Carpenter et al. 1998: 21; Thompson et al. 2012: 64, Arbour et al. in press: 52).
52. Maxillary and/or dentary tooth crown shape: ≥13 denticles, tooth crown pointed (0); <13 denticles, tooth crown rounded (1). (Thompson et al. 2012: 65, Arbour et al. in press: 53).
53. Number of dentary teeth: <25 (0); ≥25 (1). (Thompson et al. 2012: 66, Arbour et al. in press: 54).
54. Position of mandible articulation relative to mandibular adductor fossa: posterior (0); posteromedial (1). (Sereno 1999: 64; Thompson et al. 2012: 67, Arbour et al. in press: 55).
55. Mandibular fenestra: present (0); absent (1). (Thompson et al. 2012: 68).
56. Depth of the dentary symphysial ramus relative to half the maximum depth of the mandibular ramus in lateral view: deeper (0); shallower (1). (Sereno 1999: 17; Thompson et al. 2012: 69, Arbour et al. in press: 57).
57. Shape of dorsal margin of the dentary in lateral view: straight (0); sinuous (1). (Sereno 1999: 4; Thompson et al. 2012:70, Arbour et al. in press: 58).
58. Development of the coronoid process: not developed (0); distinct (1). (Sereno 1999: 108; Thompson et al. 2012: 73, Arbour et al. in press: 59**)**.
59. Position of glenoid for quadrate relative to mandibular axis: medially offset (0); in line (1). (after Carpenter et al. 1999; Thompson et al. 2012: 74, Arbour et al. in press: 60).
60. Size and projection of the retroarticular process: small with no dorsal projection (0); well developed with a dorsal projection (1). (Thompson et al. 2012: 75, Arbour et al. in press: 61).
61. Size of predentary ventral process: distinct, prong shaped process (0); rudimentary eminence (1). (Sereno 1999: 66; Thompson et al. 2012:76, Arbour et al. in press: 62).
62. Frontonasal and/or frontoparietal cranial ornamentation: absent (0) rugose, not differentiated into discrete polygons (caputegulae) (1), differentiated into discrete polygons (caputegulae) (2). (after Carpenter et al. 1999; Thompson et al. 2012: 78, Arbour et al. in press: 64).
63. A single large medial polygon of ornamentation in the parietal region: absent (0); present (1). (Thompson et al. 2012: 80, Arbour et al. in press: 65)
64. Median nasal caputegulum (located posterior to the supranarial ornamentation, on the midline of the skull): absent (0), present, hexagonal (1), present, triangular (1). (Vickaryous et al. 2004: 9, Thompson et al. 2012: 81, Arbour et al. in press: 66).
65. [Modified] Majority of frontonasal and/or frontoparietal caputegulum relief: caputegulae absent (0), caputegulae concave or flat (1), caputegulae strongly bulbous (2). (after Sullivan 1999 ; Thompson et al. 2012: 82, Arbour et al. in press: 67).
66. Projection of squamosal horns relative to the posterior margin of the dorsal surface of the skull: squamosal horns absent (0), horns do not project past posterior margin of skull in dorsal view (1), horns project past posterior margin of skull in dorsal view (2). (Thompson et al. 2012: 83, Arbour et al. in press: 68)
67. Shape of quadratojugal horn in dorsal view: quadratojugal horn absent (0), horn U-shaped, with round distal edge (1), horn triangular, with pointed distal edge (2). (Thompson et al. 2012: 85, Arbour et al. in press: 71)
68. Nuchal ornamentation (at posterior margin of skull roof): absent (0); present (1). (Vickaryous et al. 2004: 11, Thompson et al. 2012: 88, Arbour et al. in press: 72).
69. Posterior projection of the nuchal shelf: does not obscure occiput in dorsal view (0); obscures occiput in dorsal view (1). (Vickaryous et al. 2004: 12, Thompson et al. 2012: 89, Arbour et al. in press: 73).
70. Length of mandibular caputegulum with respect to the length of the mandible: ornamentation absent (0), ornamentation less than or equal to half the length (0) over three quarters the length (1). (after Carpenter et al. 1999; Thompson et al. 2012: 90, Arbour et al. in press: 74).
71. Type of articulation between the atlantal neural arch and intercentrum: open (0); fused in adult (1). (Sereno 1999: 19; Thompson et al. 2012: 92, Arbour et al. in press: 76).
72. Type of contact between the atlantal neural arches: no median contact (0); median contact (1). (Sereno 1999: 68; Thompson et al. 2012: 93, Arbour et al. in press: 77).
73. Contact between atlas and axis: articulated (0); fused (1). (Vickaryous et al. 2004: 46; Thompson et al. 2012: 94, Arbour et al. in press: 78).
74. Dimensions of cervical vertebrae centra: anteroposteriorly longer than transverse width (0); anteroposteriorly shorter than transverse width (1). (after Kirkland et al. 1998; Thompson et al. 2012: 95, Arbour et al. in press: 79).
75. Ratio of maximum neural spine width to height in anterior cervicals: <0.25 (0); ≥0.25 (1). (after Carpenter et al. 1999; Thompson et al. 2012: 96, Arbour et al. in press: 80).
76. Alignment of anterior and posterior faces of cervical centra: aligned (0); anterior face dorsal to posterior face (1); anterior face ventral to posterior face (2). (Vickaryous et al. 2004: 47, Thompson et al. 2012: 97, Arbour et al. in press: 81).
77. Ratio of anteroposterior [dorsal] centrum length to posterior centrum height: >1.1 (0); <1.1 (1). (Thompson et al. 2012: 98, Arbour et al. in press: 82).
78. Longitudinal keel on ventral surface of dorsal centra: present (0); absent (1). (Thompson et al. 2012: 99, Arbour et al. in press: 83).
79. Cross sectional shape of neural canal in posterior dorsals: circular (0) elliptical, with long axis running dorsoventrally (1). (after Carpenter 1990; Thompson et al. 2012: 100, Arbour et al. in press: 84).
80. Shape of the proximal cross-section of the dorsal ribs: triangular (0); ‘L’- or ‘T’-shaped (1). (Thompson et al. 2012: 101, Arbour et al. in press: 85).
81. Attachment of dorsal ribs to posterior dorsal vertebrae: articulated (0); fused (1). (Thompson et al. 2012: 102, Arbour et al. in press: 86).
82. Contact between posteriormost dorsal vertebrae: articulated (0); fused to form a presacral rod (1). (Thompson et al. 2012: 103, Arbour et al. in press: 87).
83. Paravertebrae: absent (0); present (1). (Thompson et al. 2012: 104, Arbour et al. in press: 88)
84. Longitudinal groove in ventral surface of the sacrum: absent (0); present (1). (Thompson et al. 2012: 105, Arbour et al. in press: 89).
85. Ratio of maximum distal width to height of the neural spines of proximal caudals: ≤0.2 (0); >0.2 (1). (after Carpenter 2001; Thompson et al. 2012: 107, Arbour et al. in press: 90**)**.
86. Direction of the transverse processes of proximal caudals: craniolaterally projecting (0); caudolaterally projecting (1); laterally projecting (2). (after Carpenter 2001; Thompson et al. 2012: 108, Arbour et al. in press: 91).
87. Persistence of transverse processes down the length of the caudal series: not present beyond the mid-length of the series (0); present beyond the mid-length of the series (1). (Thompson et al. 2012: 110, Arbour et al. in press: 92).
88. Attachment of haemal arches to their respective centra: articulated (0); fused (1). (Thompson et al. 2012: 111, Arbour et al. in press: 93).
89. Shape of distal caudal postzygapophyses: short with a sub-triangular end [wedge-shaped] (0); long with a rounded end [tongue shaped] (1). (Sereno 1999: 110; Thompson et al. 2012: 112, Arbour et al. in press: 94).
90. Extent of pre- and postzygapophyses over their adjacent centra in posterior vertebrae: extend over less than half the length of the adjacent centrum (0); extend over more than half the length of the adjacent centrum (1). (Sereno 1999: 109; Thompson et al. 2012:113, Arbour et al. in press: 95).
91. Shape of the posterior haemal arches: rounded haemal spine in lateral view with no contact between haemal arches (0); inverted ‘T’-shaped haemal spine in lateral view, with contact between the ends of adjacent spines (1). (Sereno 1999: 71; Thompson et al. 2012: 114, Arbour et al. in press: 96).
92. Ossified tendons in distal region of tail: absent (0); present (1). (Sereno 1999: 97; Thompson et al. 2012: 115, Arbour et al. in press: 97)
93. Dimensions of coracoid: longer than wide (0); wider than long or equal width and length (1). (Thompson et al. 2012: 116, Arbour et al. in press: 98).
94. Form of the anterior margin of the coracoid: convex (0); straight (1). (Thompson et al. 2012: 117, Arbour et al. in press: 99).
95. Cranioventral process of coracoid: absent (0); present (1). (Thompson et al. 2012: 118, Arbour et al. in press: 100).
96. Size of coracoid glenoid relative to scapula glenoid: sub-equal (0); half the size (1). (Sereno 1999: 89; Thompson et al. 2012: 119, Arbour et al. in press: 101).
97. Contact between scapula and coracoid: articulated (0); fused (1). (Thompson et al. 2012: 120, Arbour et al. in press: 102).
98. Scapula glenoid orientation: ventrolateral (0); ventral (1). (Sereno 1999: 87; Thompson et al. 2012: 121, Arbour et al. in press: 103).
99. Ventral process of scapula at the caudoventral margin of glenoid: absent (0); present (1). (Thompson et al. 2012: 122, Arbour et al. in press: 104).
100. Form of the scapula acromion process: not developed or ridge-like along the dorsal border of the scapula (0) tab-like, perpendicular to scapular blade (1) flange-like and folded over towards the scapula glenoid (1) ridge terminating in a knob-like eminence (2). (Vickaryous et al. 2004: 52, Thompson et al. 2012: 123, Arbour et al. in press: 105)
101. Orientation of the acromion process of scapula: directed away from the glenoid (0); directed towards scapula glenoid (1). (after Kirkland 1998; Thompson et al. 2012: 124, Arbour et al. in press: 106).
102. Scapulocoracoid buttress: absent (0); present (1). (Thompson et al. 2012: 125, Arbour et al. in press: 107).
103. Distal end of scapula shaft: narrow (0); expanded (1). (Sereno 1999: 20; Thompson et al. 2012: 126, Arbour et al. in press: 108).
104. Contact between sternal plates: separate (0); fused (1). (Sereno 1999: 112; Vickaryous et al. 2004: 60; Thompson et al. 2012: 127, Arbour et al. in press: 109).
105. Separation of humeral head and deltopectoral crest in anterior view: continuous (0); separated by a distinct notch (1). (Thompson et al. 2012: 128, Arbour et al. in press: 110).
106. Separation of humeral head and medial tubercle in anterior view: continuous (0); separated by a distinct notch (1). (Thompson et al. 2012: 129, Arbour et al. in press: 111)
107. Ratio of deltopectoral crest length to humeral length: ≤0.5 (0); >0.5 (1). (Thompson et al. 2012: 130, Arbour et al. in press: 112).
108. Orientation of deltopectoral crest projection: lateral (0); anterolateral (1). (Sereno 1999: 113; Thompson et al. 2012: 131, Arbour et al. in press: 113).
109. Shape of the radial condyle of humerus round / proximal end of radius in end-on view: non-circular (0); circular (1). (Thompson et al. 2012: 132, Arbour et al. in press: 114).
110. Ratio of the length of metacarpal V to metacarpal III: ≤0.5 (0); >0.5 (1). (Sereno 1999: 6; Thompson et al. 2012: 133, Arbour et al. in press: 115).
111. Manual digit number: 5 (0); 4 (1); 3 (2). (Thompson et al. 2012: 134, Arbour et al. in press: 116).
112. Shape of manual and pedal ungual phalanges: claw shaped (0); hoof shaped (1). (Sereno 1999: 7; Thompson et al. 2012: 135, Arbour et al. in press: 117).
113. Length of the preacetabular process of ilium as a percentage of total ilium length: ≤ 50% (0); > 50 %. (Thompson et al. 2012: 136, Arbour et al. in press: 118).
114. Angle of lateral deflection of the preacetabular process of the ilium: 10º–20º (0); 45º (1). (Sereno 1999: 21; Thompson et al. 2012: 137, Arbour et al. in press: 119).
115. Orientation of the preacetabular portion of the ilium: near vertical (0); near horizontal (1). (Kirkland 1998: 45; Thompson et al. 2012: 138, Arbour et al. in press: 120).
116. Form of the preacetabular portion of the ilium: straight process (0); pronounced ventral curvature (1). (Thompson et al. 2012: 139, Arbour et al. in press: 121).
117. Lateral exposure of the acetabulum: exposed (0) acetabulum partially obscured as it is partially encircled by the distal margin of the ilium (1). (Thompson et al. 2012: 140, Arbour et al. in press: 122)
118. Perforation of the acetabulum: present, open acetabulum (0); absent, closed acetabulum (1). (Sereno 1999: 74; Thompson et al. 2012: 141, Arbour et al. in press: 123).
119. Postacetabular ilium length, relative to diameter of acetabulum: greater (0); smaller (1). (Sereno 1999: 114; Thompson et al. 2012: 142, Arbour et al. in press: 124).
120. Shape of ischium: straight (0); ventrally flexed at mid-length (1). (Kirkland 1998: 37; Thompson et al. 2012: 147, Arbour et al. in press: 125).
121. Shape of the dorsal margin of ischium: straight or concave (0); convex (1). (Sereno 1999: 115; Thompson et al. 2012: 148, Arbour et al. in press: 126).
122. Angle between long axis of femoral head and long axis of shaft: <100º (0); 100º to 120º (1); >120º (2). (Thompson et al. 2012: 149, Arbour et al. in press: 127).
123. Separation of femoral head from greater trochanter: continuous (0); separated by a distinct notch or change in slope (1). (Thompson et al. 2012: 150, Arbour et al. in press: 128).
124. Differentiation of the anterior trochanter of the femur: separated from femoral shaft by a deep groove laterally and dorsally (0); fused to femoral shaft (1). (Kirkland 1998: 36; Thompson et al. 2012: 151, Arbour et al. in press: 129).
125. Oblique ridge on lateral femoral shaft, distal to anterior trochanter: absent (0); present (1). (Thompson et al. 2012: 152, Arbour et al. in press: 130).
126. Form of the fourth trochanter: pendant (0); ridge-like (1). (Sereno 1999: 24; Thompson et al. 2012: 153, Arbour et al. in press: 131).
127. Location of the fourth trochanter on the femoral shaft: proximal (0) distal, over half-way down the femoral shaft (1). (Thompson et al. 2012: 154, Arbour et al. in press: 132).
128. Maximum distal width of the tibia, compared to the maximum proximal width: narrower (0); wider (1). (Sereno 1999: 188; Thompson et al. 2012: 155, Arbour et al. in press: 133).
129. Contact between tibia and astragalus: articulated (0); fused, with suture obliterated (1). (Thompson et al. 2012: 156, Arbour et al. in press: 134).
130. Number of pedal digits: 5 (0); 4 (1); 3 (2). (Thompson et al. 2012: 157, Arbour et al. in press: 135).
131. Phalangeal number in pedal digit IV: 5 (0); ≤4 (1). (Sereno 1999: 26; Thompson et al. 2012: 158, Arbour et al. in press: 136).
132. Parasagittal row of keeled osteoderms situated on the dorsal aspect of the trunk: absent (0); present (1). (Sereno 1999: 2; Thompson et al. 2012: 159, Arbour et al. in press: 137).
133. Number of distinct cervical pectoral bands: none (0); one (1); two (2). (Kirkland 1998: 38; Thompson et al. 2012: 162, Arbour et al. in press: 138).
134. Form of sacral armour: unfused (0), coossified osteoderm rosettes (1), coossified evenly-sized polygons (2). (Arbour et al. in press: 140)
135. Small (<2 cm diameter), circular osteoderms posterolateral to orbit, along ventral edge of squamosal horn and/or along dorsal edge of quadratojugal horns: absent (0); present (1) (Arbour et al. in press: 142)
136. Form of cervical half rings: cervical half rings absent (0), composed of osteoderms that are either tightly adjacent to one another or coossified at the edges, forming arc over the cervical region (1), composed of osteoderms and underlying bony band segments, osteoderms may or may not cossify to the band, forming arc over the cervical region (2). (Arbour et al. in press: 143)
137. Composition of first cervical half ring with band: no cervical half ring with band (0), first cervical half ring has 4 to 6 primary osteoderms only (1), first cervical half ring has 4 to 6 primary osteoderms surrounded by small (<2 cm diameter) circular secondary osteoderms (2). (Arbour et al. in press: 144)
138. Form of caudal osteoderms: dorsoventrally compressed, triangular in dorsal view (0), or low cones (1). (Arbour and Currie 2013:174, Arbour et al. in press: 145)
139. Tail club knob shape: knob absent (0), major knob osteoderms semicircular in dorsal view (1), triangular in dorsal view (2). (Arbour and Currie 2013:175, Arbour et al. in press: 146)
140. Tail club knob proportions: knob absent (0), tail club knob length > width (1), length = width (2), width > length (3) (Arbour and Currie 2013:176, Arbour et al. in press: 147)
141. Shape of respiratory passage: straight or arched (0), with anterior (rostral) and posterior (caudal) loops (sensu Witmer and Ridgely 2008). (Arbour et al. in press: 148)
142. Lacrimal incisure (mediolateral constriction behind the narial osteoderms/at the prefrontals, giving the skull an hourglass-shaped outline in dorsal view): absent (0) present (1) (Arbour et al. in press: 149)
143. Domed frontonasal caputegulae: domed caputegulae absent (0), rounded cones with circular bases (1) pyramidal with sharp edges (2) (Arbour et al. in press: 150)
144. Number of internarial caputegulae: none (0), 1 (1), more than 1 (2). (Arbour et al. in press: 151)
145. Supranarial caputegulae, notch dorsal to nasal vestibule: no caputegulae (0), notch absent (1), notch present (2). (Arbour et al. in press: 152)
146. Loreal caputegulum in lateral view: no caputegulum (0) 1 caputegulum (0), more than 1 caputegulum (1) (Arbour et al. in press: 153)
147. Lacrimal caputegulum in lateral view: no caputegulum (0) 1 caputegulum (0), more than 1 caputegulum (1) (Arbour et al. in press: 154)
148. Prefrontal caputegulum: no caputegulum (0) small, flat (1), prominent, sharply pointed and pyramidal (2). (Arbour et al. in press: 155)
149. Depth of jugal ramus relative to orbit height: jugal height is less than 15% orbit height (0), jugal height is more than 15% orbit height (1) (Arbour et al. in press: 156)
150. Supraorbital caputegulae, when viewed dorsally: no caputegulae (0), combine to form continuous edge (1), have distinct apices (2). (Arbour et al. in press: 157)
151. Accessory postorbital ossification: absent (0), present (1) (Arbour et al. in press: 158)
152. Quadratojugal horn: no horn (0) lacks distinct neck at base (1), has distinct neck at base (2). (Arbour et al. in press: 159)
153. Squamosal horn: no horn (0) base has broad triangular cross-section and overall shape is pyramidal (1), base is oval in cross-section and overall shape is narrow, tapered cylinder (2) (Arbour et al. in press: 160)
154. New character: Number of caputegulae in frontonasal and prefrontal region: no caputegulae (0), 10 or fewer (1), 11 to 30 (2) more than 30 (3)

**Part 3: Literature Cited**

Arbour VM, Currie PJ, Badamgarav D (In press) The ankylosaurid dinosaurs of the Upper Cretaceous Baruungoyot and Nemegt formations of Mongolia. Zoological Journal of the Linnean Society.

Arbour VM, Currie PJ (2013) *Euoplocephalus* *tutus* and the diversity of ankylosaurid dinosaurs from the Late Cretaceous of Alberta, Canada, and Montana, USA. PLOS ONE 8:e62421,

Carpenter, K. 1990. Ankylosaur systematics: an example using *Panoplosaurus* and *Edmontonia* (Ankylosauria: Nodosauridae). In: Carpenter K, Currie PJ, editors. Dinosaur Systematics: Perspectives and Approaches. Cambridge: Cambridge University Press, pp. 281–297.

Carpenter, K. 2001. Phylogenetic analysis of the Ankylosauria. In: Carpenter K, editor. The Armoured Dinosaurs. Bloomington: Indiana University Press, pp. 455–483.

Carpenter K, Miles C, Cloward K (1998) Skull of a Jurassic ankylosaur (Dinosauria). Nature 393: 782–783.

Carpenter K, Kirkland JI, Burge D, Bird J (1999) Ankylosaurs (Anklosauria: Ornithischia) of the Cedar Mountain formation, Utah, and their stratigraphic distribution. In: Gillette DD, editor. Vertebrate Paleontology in Utah, Utah Geological Survey Miscellaneous Publications 9. Salt Lake City: Utah Geological Survey, pp. 243–251.

Hill RV, Witmer LM, Norell MA (2003) A new specimen of *Pinacosaurus* *grangeri* (Dinosauria: Ornithischia) from the Late Cretaceous of Mongolia: ontogeny and phylogeny of ankylosaurs. American Museum Novitates 3395:1–29.

Kirkland JI (1998) A polacanthine ankylosaur (Ornithischia: Dinosauria) from the Early Cretaceous (Barremian) of eastern Utah. New Mexico Museum of Natural History and Science Bulletin 14:271–281.

Kirkland JI, Carpenter K, Hunt AP, Scheetz RD (1998) Ankylosaur (Dinosauria) specimens from the Upper Jurassic Morrison Formation. Modern Geology 23:145-177.

Lee Y-N (1996) A new nodosaurid ankylosaur (Dinosauria: Ornithischia) from the Paw Paw Formation (Late Albian) of Texas. Journal of Vertebrate Paleontology 16:232–245.

Sereno PC (1999) The evolution of dinosaurs. Science 284:2137–2147.

Sullivan RM (1999) *Nodocephalosaurus* *kirtlandensis*, gen. et sp nov., a new ankylosaurid dinosaur (Ornithischia: Ankylosauria) from the Upper Cretaceous Kirtland Formation (Upper Campanian), San Juan Basin, New Mexico. Journal of Vertebrate Paleontology 19: 126–139.

Thompson RS, Parish JC, Maidment SCR, Barrett PM (2012) Phylogeny of the ankylosaurian dinosaurs (Ornithischia: Thyreophora). Journal of Systematic Palaeontology 10: 301-312.

Vickaryous MK, Russell AP, Currie PJ, Zhao X (2001) A new ankylosaurid (Dinosauria: Ankylosauria) from the Lower Cretaceous of China, with comments on ankylosaurian relationships. Canadian Journal of Earth Sciences 38:1767–1780.

Vickaryous MK, Maryańska T, Weishampel DB (2004) Ankylosauria. In: Weishampel DB, Dodson P, Osmólska H, editors. The Dinosauria, 2^nd^ edition. Berkeley: University of California Press, Berkeley, pp. 363–392.

Witmer LM, Ridgely RC (2008) The paranasal air sinuses of predatory and armored dinosaurs (Archosauria: Theropoda and Ankylosauria) and their contribution to cephalic structure. The Anatomical Record 291:1362-1388.
